# Holistic View on the Structure of Immune Response: Petri Net Model

**DOI:** 10.3390/biomedicines11020452

**Published:** 2023-02-04

**Authors:** Sonja Scharf, Jörg Ackermann, Leonie Bender, Patrick Wurzel, Hendrik Schäfer, Martin-Leo Hansmann, Ina Koch

**Affiliations:** 1 Molecular Bioinformatics, Institute of Computer Science, Goethe University Frankfurt, Robert-Mayer Str. 11-15, 60325 Frankfurt am Main, Germany; 2 Frankfurt Institute for Advanced Studies, Ruth-Moufang-Str. 1, 60438 Frankfurt am Main, Germany; 3 Institute of General Pharmacology and Toxicology, Goethe University Frankfurt, Theodor-Stern-Kai 7, 60590 Frankfurt am Main, Germany; 4 Institute of Pathology, Corporate Member of Free University of Berlin, Humboldt-University of Berlin, Charity-University Medicine Berlin, Virchowweg 15, 10117 Berlin, Germany

**Keywords:** Petri net, lymph node, simulation, antigen, B-cell differentiation, invariant

## Abstract

The simulation of immune response is a challenging task because quantitative data are scarce. Quantitative theoretical models either focus on specific cell–cell interactions or have to make assumptions about parameters. The broad variation of, e.g., the dimensions and abundance between lymph nodes as well as between individual patients hampers conclusive quantitative modeling. No theoretical model has been established representing a consensus on the set of major cellular processes involved in the immune response. In this paper, we apply the Petri net formalism to construct a semi-quantitative mathematical model of the lymph nodes. The model covers the major cellular processes of immune response and fulfills the formal requirements of Petri net models. The intention is to develop a model taking into account the viewpoints of experienced pathologists and computer scientists in the field of systems biology. In order to verify formal requirements, we discuss invariant properties and apply the asynchronous firing rule of a place/transition net. Twenty-five transition invariants cover the model, and each is assigned to a functional mode of the immune response. In simulations, the Petri net model describes the dynamic modes of the immune response, its adaption to antigens, and its loss of memory.

## 1. Introduction

In contrast to the human neural system, which is centrally coordinated by the brain, the immune system is decentralized. The lymph nodes are important compartments of the immune system. Every human organism has about 600 lymph nodes, which control and defend the different areas and organs, located at strategically important positions. Lymph nodes are perfused by lymph, which contains many substances and different forms of antigens. The lymph nodes show afferent and efferent lymphoid and blood vessels. One of the aims of the lymph nodes is to clean the body of internal and external antigens and apoptotic cells. The spatial differentiation of the lymph node and the concerted action of various cell types, e.g., “effector” T cells and “memory” B cells, are powerful strategies that enable the human body to survive the attacks of bacteria, viruses, and tumor cells.

The lymphatic system and immune response have been extensively studied by mathematical modeling. For an introduction and review, we refer to Margaris and Black [1] and Cappuccino et al. [2], respectively. Mathematical models involving the lymph nodes have covered various levels of abstraction, ranging from the molecular signaling pathways to whole-body models [3,4,5,6]. Appropriate modeling techniques include, among many others, classical ordinary differential equations (ODEs), agent-based models [7], Potts models, graph theory-based models, Petri nets (PNs), and Boolean networks. Early ODE models have been presented, e.g., by Kesmir and De Boer [8] for the germinal center kinetics and by Miao et al. [9] for the adaptive immune response. Swerdlin et al. [10] applied “the visual language of Statecharts” to model the lymph node B-cell immune response. Their model describes compartmentalized lymph node and cell–cell interactions. A computational model of the 3D geometry of the lymph node (LN) as been presented by Kislitsyn et al. [11]. Pennisi et al. [12] have proposed a methodological approach based on colored PNs to model the immune system at the cellular scale. A PN model of the adaptive immune system with signaling proteins during a hepatitis C virus infection has been proposed by Obaid et al. [13]. Thomas et al. [14] have developed an extension of the Gillespie algorithm to simulate the germinal center reaction. Pernice et al. [15] have adopted PN techniques to simulate the stochastic reaction kinetics of the immune response in multiple sclerosis. PNs have been applied to model biological systems such as inflammation and oxidative stress [16] and the influence of micro-environmental signals on macrophage differentiation [17].

A PN is a mathematical model for representation and analysis of concurrent processes [18]. The problem of whether some arbitrary state is reachable from a fixed initial state of a PN is of basic importance in the field of computer science [19,20,21,22,23,24,25]. PNs enable modeling in a multi-modal way, ranging from semi-quantitative to detailed quantitative modeling. PNs offer a solid mathematical foundation, analysis and simulation, intuitive visualization, and a number of valuable user-friendly tools. PNs have a broad application in the modeling of biological systems [26,27,28]. PNs have been extended with fuzzy logic approaches in the context of gene regulatory network modeling [29], and have been combined with ODEs to model signaling networks [30]. In the field of biological PN models, the analysis of invariants has proven valuable in application such as gene regulation [31], signal transduction [32,33,34,35,36], and metabolic networks [37,38]. Place invariants (PIs), transition invariants (TIs, also known as elementary modes), and Manatee invariants (MIs) have been demonstrated to provide valuable insights into the function and pathways of biological networks. The properties of a network to be covered by transition invariants (CTI) or place invariants (conservative networks) are helpful in verifying the model, especially during the network construction phase. For metabolic models and signal transduction models, the benefit of invariants arises from their biological interpretation. For cellular systems, the concept of invariant properties or related concepts, e.g., in silico knockout [36], have not been evaluated.

A perfect theoretical model would quantitatively simulate the immune response of individual patients, adaption to pathogens (e.g., variants of the severe acute respiratory syndrome coronavirus type 2 (SARS-CoV-2) [39]), the role of specific diseases (e.g., nodular sclerosing Hodgkin lymphoma, which destroys the structural integrity of lymph nodes [40]), and the treatment of patients with engineered T cells [41]. Currently, ongoing digitization in clinical laboratories and pathology is leading to the aggregation of more and more data. The development of a quantitative “virtual lymph node” may become possible. The first step in the direction of a quantitative model is agreement on its structure and the set of major processes that have to be included. A prerequisite for feed-in of quantitative data is a holistic view of the structure of the immune response. PN techniques are well suited for the task, because modeling requires no quantitative data, rigorous methods for verification exist, and intuitive visualization supports the cooperation between computer scientists and clinicians.

**Figure 1:**
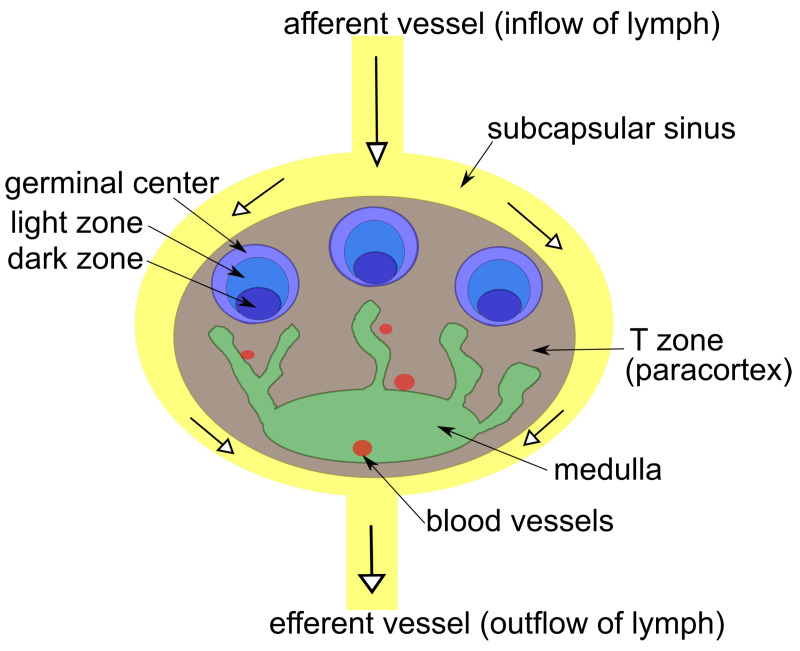
Sketch of a lymph node as a structured organ with compartments: blood vessels (red), subcapsular sinus (yellow), medulla (green), T zone (brown), and germinal center (light blue) with light zone (blue) and dark zone (dark blue). Lymph enters the subcapsular sinus via the topafferent vessel, flows around the lymphoid compartments, and may enter the T zone.

Here, we present a PN model of the lymph node (Figure 1), representing the concerted effort of theoretical modelers and clinical researchers. This model enables evaluation and verification with rigorous methods from computer science. The PN model opens up the possibility of studying central problems of computer science, e.g., invariant properties, in silico knockout, and reachability, for the case of the lymph node. Note that the lymph node processes information about invading pathogens in order to adapt its immune response, and can itself be seen as an information processing unit. From a biological viewpoint, the PN provides a holistic view of the immune response of the lymphatic system. Quantitative models on specific processes may be superior in answering specific questions. For the development of a holistic quantitative model, however, the PN may be the best starting point.

We tested approaches that had previously been conventionally applied only for molecular systems, such as metabolic systems and signal transduction systems. Here, we provide a biological interpretation of all invariant properties. We propose a workflow for systematically testing and verifying the network model during the construction phase. We formulate a set of structural requirements that a biologically meaningful PN model of cellular systems must fulfill. We perform an in silico knockout analysis of the model. Our simulations of the model were in accordance with the current understanding of the dynamics of an immune response, without using any kinetic data. The PN model enables further experiments to be run in order simulate hypotheses and generate new hypotheses.

## 2. Materials and Methods

**Figure 2:**
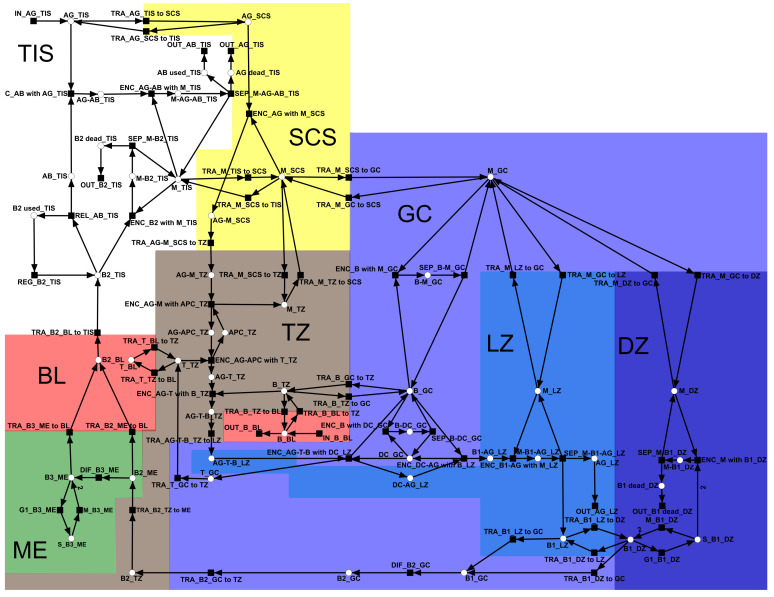
Petri net model of a lymph node. Rectangles, circles, and arcs represent transitions, places, and edges, respectively. Places denote cells, e.g., B cells, T cells, dendritic cells, and macrophages. Transitions denote cell interaction, activation, and migration. The lymph node (LN) is structured into the T zone (TZ, brown), germinal center (GC, blue), subcapsular sinus (SCS, yellow), medulla (ME, green), light zone (LZ, light blue), and dark zone (DZ, dark blue). Cells may enter and leave the lymph node via blood (BL, red) or tissue (TIS, not highlighted). For lists of places and transitions, refer to Table A1 and Table A2 in Appendix A, respectively. For the reaction system, refer to Table A3 in Appendix A.

A PN is a bipartite-directed weighted graph. The two types of nodes are called places and transitions; *P* and *T* denote finite sets of places and transitions, respectively. In our graphical representations (see Figure 2), places and transitions are represented by circles and squares, respectively. The set of arcs F⊆(P×T)∪(T×P) describes connections from places to transitions or vice versa. Each arc f∈F has a nonzero integer weight w(f)∈N . Each place p∈P carries an integer number marking $m\left(p\right)\in N_{0}$ for the quantity of so-called tokens $m_{0}\left(p\right)\in N_{0}$ , where $m_{0}$ is called the initial marking. In mathematical terms, a PN is defined as a quintuple $N=(P,T,F,W,m_{0})$ with:

- a finite set of places *P*
- a finite set of transitions *T* that are disjunctive from *P*, T∩P=∅
- a set of arcs F⊆(P×T)∪(T×P)
- the weights of arcs W:f∈F→w(f)∈N
- an initial marking $m_{0}:p\in P\to m_{0}\left(p\right)\in N_{0}$ .

For each transition *t*, the set of arcs *F* defines a set of pre-places •t={p|(p,t)∈F} and a set of post-places t•={p|(t,p)∈F} . Border transitions are transitions without post-places (output transitions) or pre-places (input transitions). Tokens are movable objects. The number of tokens on the places, i.e., the marking, can change according to a firing rule. Here, we apply the classical P/T (place/transition) firing rule. A transition is allowed to fire, i.e., is activated or has a concession, if and only if a sufficient number of tokens is located on all of its pre-places. The firing of transition *t* removes a number of tokens from each pre-place p∈•t according to the weight w(f) of arc f=(p,t) . Simultaneously, the firing of transition *t* adds tokens on each post-place p∈t• according to the weight w(f) of arc f=(t,p) .

A PN is called *pure* if no transition has a pre-place that is also a post-place. A PN is *connected* if an undirected path (ignoring the direction of arcs) exists between each pair of nodes. A PN is *strongly connected* if a directed path exists between each pair of nodes. A PN is called *ordinary* if all arcs have weights of one. A PN is *conserved* if the sum of tokens in all places does not change by the firing of transitions.

Each entry $c_{ij}$ of the *incidence matrix* *C* denotes the change of tokens to place *i* if a transition *j* fires. The number of occurrences of transition *i* in a firing sequence *s* of transitions is provided by the component $x_{i}=\#i$ of a vector $x:T\to N_{0}$ . In context-free grammars and Petri nets, the vector *x* is called the *Parikh vector* of sequence *s*. The minimal set of Parikh vectors *x* that satisfy the equation C×x=0 is called *TI*. If all transitions are *covered by TIs*, the PN is called *CTI*. The minimal set of semi-positive integer vectors *y* that satisfy the equation y×C=0 is called *PI*. If all places are covered by PIs, the PN is *covered by place invariants (CPI)*. *MIs* are linear combinations of TIs that aim to be feasible for the empty initial marking. For a detailed definition and discussion, readers are referred to Amstein et al., 2017 [35].

We applied the software MonaLisa [42] to construct, visualize, and analyze our PN model. To determine various mathematical properties of the PN, we applied the INA tool [43,44]. For the in silico knockout, we applied the software isiKnock [33].

## 3. Results

We constructed a PN model of the lymph node. We focused on the interaction of cells in the compartments of the lymph node. Properties of the model such as PI, TI, and MI, were analyzed. In addition, we performed an in silico knockout analysis and simulations.

### 3.1. Construction

We constructed a PN model of cell migration, cell–cell interactions, cell activation, cell differentiation, and cell proliferation inside a lymph node. In the model, the lymph node (LN) is a structured organ with a subcapsular sinus (SCS), paracortex (TZ for T Zone), medulla (ME), and germinal center (GC) with light (LZ) and dark zone (DZ). For illustration and a list of abbreviations of compartments, refer to Figure 1 and Table 1, respectively. Inside the LN model, various cell types are responsible for the immune response: T cells, B cells, macrophages, follicular dendritic cells (DCs), and antigen-presenting cells (APCs). For a list of cells and their abbreviations, see Table 2. For an introduction to the architecture of LNs and cellular traffic in LNs, interested readers are referred to [45].

We constructed the PN model from scratch, starting with hand drawings by an expert pathologist in our group. The expert pathologist had decades of experience in diagnosis based on stained whole-slide images of LN and experimental research on the structure of LN, cell motility, cell–cell interaction, and cell properties; see [46,47,48,49,50,51,52,53,54,55,56,57,58,59] and citations therein. For a list of processes and their abbreviations, refer to Table 3. Our intention was to construct a basic model that considers only a minimal set of the major cellular players and compartments of the LN. The model was indented to mimic an adaptive immune response in accordance with the current understanding of the LN dynamicswithout using any kinetic data, as these are not available in most cases. For the analysis of the PN model of the LN, we constructed a PN without border transitions with an arc weights of one. Thus, the model represents a place-bordered and ordinary PN (PBOPN).

We applied the following two formal conditions for the construction of a biologically meaningful PN model of the lymph node:

1. The PN is CTI. Each transition invariant has a specific biological meaning.
2. Each place invariant of a PBOPN has a specific biological meaning. A detailed definition is provided below in Section 3.3.

Formal condition (1) is the completeness property of the model. Each transition has to contribute to the function of the model, i.e., has to be an active part of a functional biological module. TIs, well-known as elementary flux modes in systems biology, represent functional modules and pathways of biochemical systems [37,60,61,62]. A transition that is not part of any TI is either unnecessary or indicates missing reactions [26,63,64]; hence, a biologically meaningful PN model should be CTI, expressing completeness.

Formal condition (2) guarantees a proper balance of the cells in the system. The total number of cells may change by cell death, cell replication, or the inflow and outflow of cells from and to the environment. We modeled the death, inflow, and outflow of cells by border transitions. Without border transition, processes such as death, inflow, and outflow would not be considered in the model. The PBOPN has no border transitions. To model cell replication, we introduced arcs with weights of two, e.g., reactions in the form of cell→2cells . The PBOPN has only arcs of weight one, and consequently lacks cell replication. In the PBOPN, the total number of cells is conserved. Special attention was required for the antibodies that are repeatedly produced by activated B cells. To keep the number of all species constant, we had to delete the production of antibodies. We required that the PBOPN without production of antibodies be conservative. This meant that each species had to be assigned to a PI describing its conservation. During the construction of the PN model, we found this check of the balance of cells and antibodies by the PBOPN to be worthwhile for the identification of biologically questionable or erroneous reactions.

### 3.2. The Petri Net Model

Figure 2 shows the full PN model of the lymph node. The PN has 49 places, 65 transitions, and 149 arcs. For a list of the places and transitions, along with their names and descriptions, refer to Table A1 and Table A2, respectively, in Appendix A. Table A3 in Appendix A lists the complete reaction system. The file Data S1 in Supplementary Material represents the PN model in SBML (Systems Biology Markup Language) [65]. The background colors in Figure 2 indicate the compartments: T zone (brown), germinal center (blue), subcapsular sinus (yellow), medulla (green), light zone (light blue), dark zone (dark blue), blood (red), and tissue (white). For a list of compartments and their abbreviations, refer to Table 1. For the nomenclature of places and transitions, we applied an encoding based on the sub-strings in Table 2 and Table 3, respectively.

In terms of PN formalism [43,44], the PN is pure, CTI, and connected. The PN has transitions without pre-places (input transitions) and transitions without post-places (output transitions). The PN has no places without pre-transition or post-transition. The PN is neither strongly connected, CPI, bounded, ordinary, or conserved.

Places represent species, e.g., B cells, T cells, dendritic cells, APC, macrophages, antigens, and antibodies. Transitions describe various processes e.g., cell–cell interaction, modification, translocation, differentiation, cell–cell contacts, activation, replication, influx, outflow, regeneration, release, and separation.

The network describes the immune response of the lymph node to an influx of antigens. Antigen (AG) enters the system via the tissue (TIS) (see input transition IN_AG_TIS in the upper left part of Figure 2). Within the lymph, the antigen flows into the subcapsular sinus (SCS) of the lymph node (see the yellow-highlighted part of Figure 2). In the SCS, macrophages (M) recognize the exogenous invader, bind to the antigen, and translocate with the AG to the T zone (paracortex) (see the brown-highlighted part of Figure 2). In the T zone, antigen-presenting cells (APC) recognize the AG and activate T cells (T). Activated T cells bind to B cells and translocate to the light zone (LZ); see the light blue-highlighted part of Figure 2. In the light zone, the T cells are stripped off and activated B cells (B1), called centroblasts, translocate to the dark zone and proliferate [66,67] (see the dark blue-highlighted part of Figure 2). From the dark zone, the newly produced activated B cells (B2), known as plasma B ells, translocate via the germinal center (blue), T zone (brown), medulla (green), and blood (red) to the tissue. In the medulla (ME), plasma B cells can be stored as memory B cells (B3) and proliferate. In the tissue, the plasma B cells produce antibodies (AB). The produced AB specifically recognizes the AG and triggers the degradation of AG by macrophages in the tissue.

### 3.3. Place Invariants of a Place-Bordered Ordinary PN

To construct the PBOPN model, we set all arc weights to one and deleted all input and output transitions. Table A1 in Appendix A shows the PBOPN. The Data S2 file in the Supplementary Material presents the PBOPN model in SBML (Systems Biology Markup Language) format. The PN has 49 places, 57 transitions, and 141 arcs. Table A7 in the Appendix A lists the ten PIs of the PN. These PIs describe the conservation of T cells (PI-0), antigen-presenting cells (PI-1), dendritic cells (PI-2), macrophages (PI-3), B cells (PI-4), activated cells (PI-5, PI-6), non-activated B cells, B cells with antigen (PI-7), and B cells with antigens PI-8 and PI-9.

The PN was not conservative, i.e., not covered by PI. Place AB_TIS was the only place not covered by a PI. Thus, the PN was not conservative. Tokens on place AB_TIS represented antigens that were newly produced by activated B cells in the tissue. Deletion of the arc to the place AB_TIS would have generated two additional PIs that represented the conservation of antibodies in the tissue.

Each PI of the PBOPN has a specific biological meaning. Each cell type could be assigned to at least one PI that described its conservation. No cells disappeared from the PN. Two pairs of PIs, (PI-5, PI-6) and (PI-8, PI-9), were identical except for a single place. Each pair described the conservation of a single species. The two redundancies of the PIs resulted from the functional coupling of species in certain reactions, e.g., the coupling between consumption of antigens and elimination of antibodies in the tissue; see transition SEP_M-AG-AB_TIS. Checking the cell balance by PI enabled control and discussion of the specific reactions for inflow and outflow, production, and elimination of species.

### 3.4. Invariants of the Full PN Model

In contrast to its PBOPN, the full PN had two input transitions and six output transitions. The two input transitions, IN_AG_TIS and IN_B_BL, described the infection of tissue with antigen and release of B cells from the bone marrow to the bloodstream, respectively. The six output transitions described the removal of species from the system, e.g., elimination of antigen, consumption of antibody, and death of B cells. The PN was not ordinary and had three arcs of weight two. One arc of weight two described the mitosis of activated B cells (B1) in the dark zone; see transition M_B1_DZ in the right bottom part of Figure 2. A second arc of weight two described mitosis memory B cells (B3) in the medulla; see transition M_B3_ME in the left bottom part of Figure 2. The third arc of weight two described the elimination of activated B cells (B1) by macrophages in the dark zone; see transition ENC_M_with_B1_DZ in the left bottom part of Figure 2.

#### 3.4.1. Place Invariants

The full PN had six fewer PIs than its PBOPN. Antigens, cells that were activated by antigens, B cells, non-activated B cells, and B cells with antigens were no longer conserved. The four remaining PIs of the full PN describe the conservation of macrophages, T cells, dendritic cells, and antigen-presenting cells, respectively; see Table A4.

#### 3.4.2. Transition Invariants

**Figure 3:**
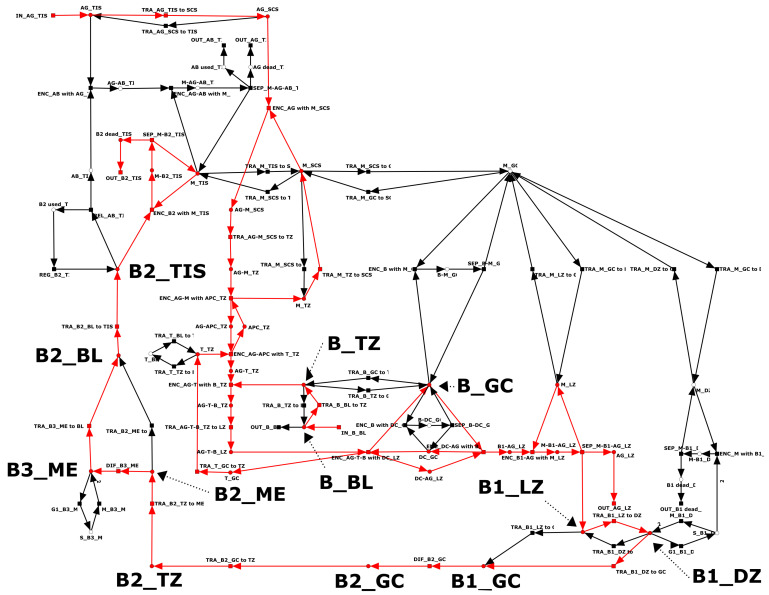
Exemplary transition invariant TI-8. The 28 transitions of TI-8 are highlighted in red. TI-8 characterizes a possible pathway of B cell activation induced by the invasion of antigens. B cells may move from the blood (place: B_BL) to the T zone (place: B_TZ) and then, after activation, pass via the light zone, dark zone, germinal center, T zone, medulla, and blood to the tissue (places: B1_LZ, B1_DZ, B1_GC, B2_GC, B2_TZ, B2_ME, B3_ME, B2_BL, B2_TIS). B cells differentiate in several steps to become activated, and eventually become degraded in the tissue. For the abbreviations used to name the places, refer to Table 1 and Table 2. For a list of TIs, refer to Table A5.

**Figure 4:**
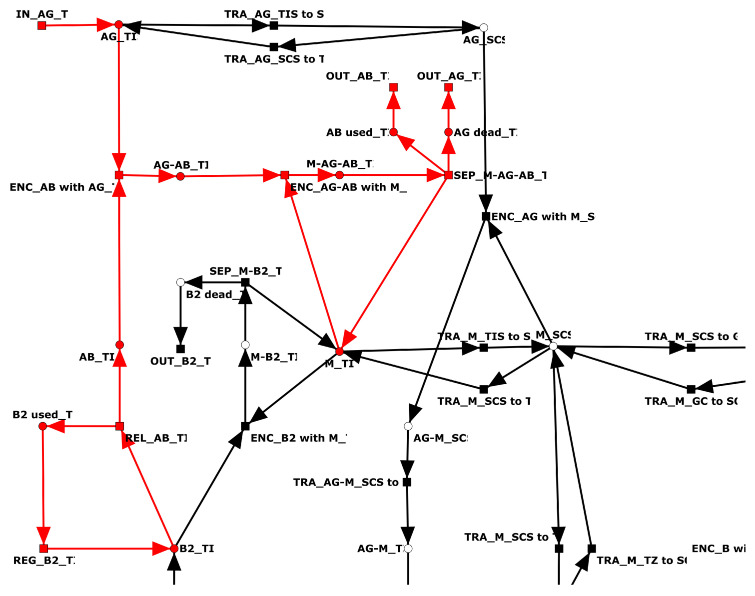
Transition invariant TI-9: production of antibodies. TI-9 is highlighted in red. TI-9 characterizes the production of antibodies (place: AB_TIS) by plasma B cells (place: B2_TIS) and the degradation of antigen (place: AG_TIS) in the tissue by macrophages (place: M_TIS).

The full PN is CTI. Table A5 in the Appendix A lists the 25 TIs which cover the network. The entry in the “length” column provides the number of transitions of the TI. The majority of TIs (*n* = 13) are trivial, i.e., they contain only two transitions. The twelve non-trivial TIs contain between five and 28 transitions. As an example, Figure 3 highlights the largest TI, with 28 transitions. TI-8 represents the activation of a B cell by an AG, its migration from the blood to the para-cortex, and its subsequent passage via the light zone, dark zone, medulla, and blood to the tissue. Within the compartments, the B cell differentiates to a centroblast (place: B1_GC), a plasma B cell (place: B2_GC), and a memory B cell (place: B3_ME). In the pathway of TI-23, the differentiated B cell is neither replicated nor does it produce any AB. Without contributing to the adaptive immune response, the B cell is finally degraded as a plasma B cell in the tissue. TI-0, TI-10, and TI-23 are very similar pathways, each with small variations in the migration of the B cell. B cells produced by replication in either the dark zone (TI-16, TI-21, TI-22, TI-24) or the medulla (TI-14) may be degraded in the tissue. Additional degradation of activated B cells may take place in the dark zone. TI-5 corresponds to the steps from activation of B cells to degradation in the dark zone. TI-20 corresponds to the replication and degradation of a B cell in the dark zone. TI-9 corresponds to the production of AB in the tissue. The eight transitions of TI-9 represent the production of AB by plasma B cells in the tissue, recognition of the AG by the AB, and the destruction of the AG by macrophages; see Figure 4.

Note that each TI corresponds to a functional module of the immune response. However, the TIs do not offer a holistic view of the adaptive immune response. Only one TI, TI-9, represents the production of AB. The transitions of TI-9, however, include neither activation of B cells by invading AG nor any replication of activated B cells in the dark zone or the medulla. Essential couplings of the activation of B cells and the replication of activated B cells with the production of AB are not reflected by functional modules determined by TIs. A similar shortcoming of the concept of TI has been observed and discussed in relation to PN models of signal transduction systems. For PN models of signal transduction systems, the concept of MI has been shown to be advantageous [35].

#### 3.4.3. Manatee Invariants

**Figure 5:**
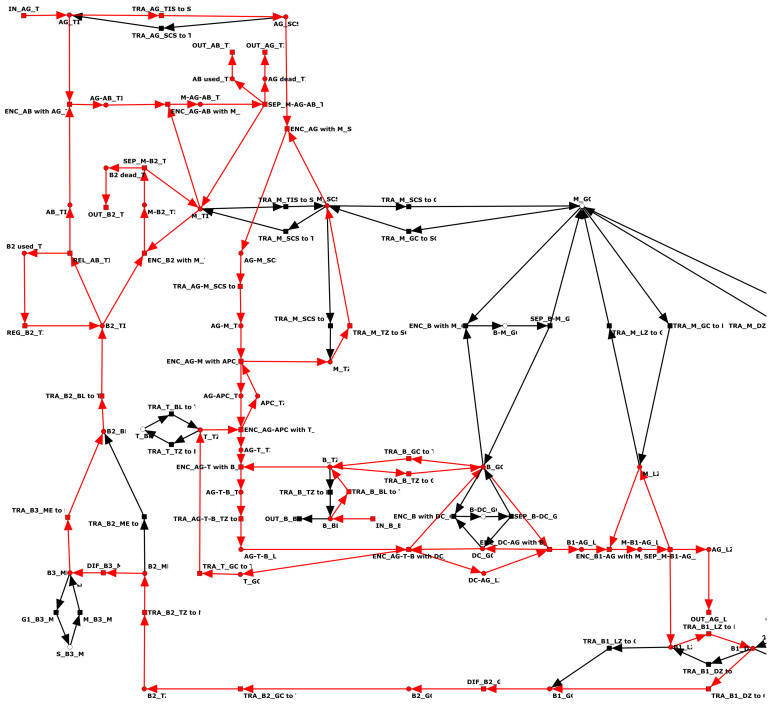
Manatee invariant MI-33: invasion of antigen leads to the activation of B cells, the production of antibodies, and the degradation of antigens. MI-33 is highlighted in red. Manatee invariant MI-33 characterizes a possible pathway of the immune response induced by the invasion of antigens and the activation of B cells. MI-33 combines transition invariants TI-6 and TI-8 (see Figure 3) as well as TI-9 (see Figure 4). For a complete list of Manatee invariants, refer to Table A6.

Table A6 in Appendix A lists the 66 MIs, the number of transitions of the MIs, the names of the TIs in the MIs, whether the MIs are pure, and whether ABs are produced. The numbers of transitions vary between two and 37 transitions. Three MIs are pure and 14 MIs release AB. Seven MIs are trivial, each of which corresponds to a single TI. The 59 non-trivial MIs are linear combinations of two to four TIs. The file Data File S3 in the Supplementary Material lists the detailed Parikh vector of each MI. Of the 14 MIs that release AB, four include the activation of B cells by invading AG, eight include the replication of activated B cells in the dark zone, and two include the replication of memory B cells in the medulla. Further, 30 MIs eliminate invading AG without any production of ABs. Of the remaining 15 non-trivial MIs, eleven MIs describe the replication of B cells and four MIs describe the migration of B cells without any elimination of the AG. Exemplary, Figure 5 highlights the largest MI, MI-33, with 37 transitions.

The majority of MIs in Table A6 in the Appendix A were impure. Only three MIs were pure: MI-2, MI-5, and MI-19. These three MIs describe the production and degradation of B cells in the blood in combination with the free movement of B cells to and from the T zone and germinal center. Neither an activation nor any interaction with other cells or antigens is involved in these pure MIs. This means that all activation processes and functional modules of the immune response are described by impure MIs. Each impure MI induces a subnet that contains at least one PI. The feasibility of impure MIs relies on the necessary condition of sufficient tokens on these PI; hence, none of the impure MIs can be feasible for a PN with empty initial marking.

#### 3.4.4. Prediction of System Behavior via in Silico Knockout

**Figure 6:**
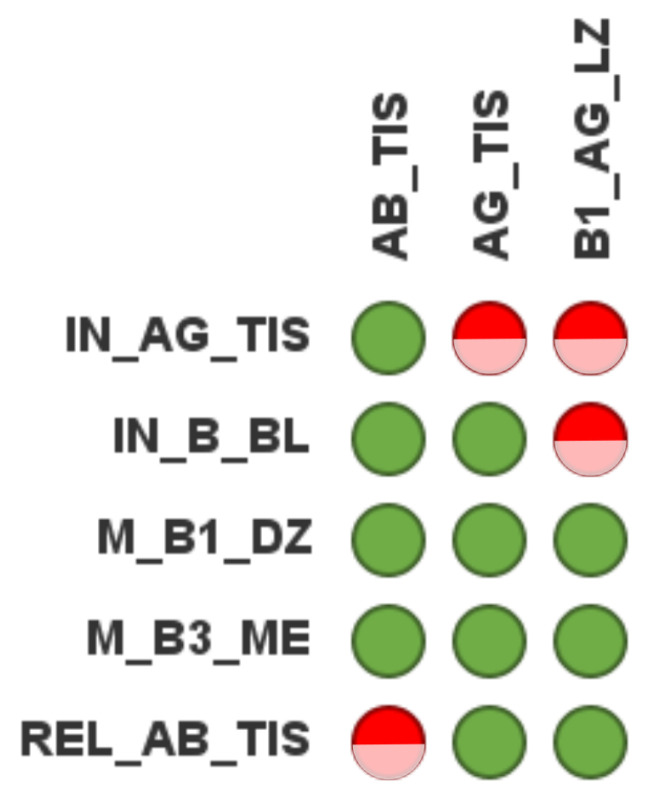
Example knockout matrix. The name of a knocked out transition is indicated by the label of the row. The name of an affected place is indicated by the label of a column. Half-filled red circles denote that the knockout had an effect; green circles denote no effect of the knockout. The knockout matrix is computed by an in silico knockout based on Manatee invariants [36]. We chose five transitions and three places. The knockout of transition IN_AG_TIS (first row) stops the invasion of antigens. No antigen enters the tissue (red circle for AG_TIS) and no activated B cell enters the light zone (red circle for B1_AG_LZ), though the production of antibodies may continue to be active (green circle for AB_TIS). Overall, the majority of circles are green (no effect of a knockout). The knockout matrix indicates the robustness of the immune response to single knockouts; see text.

Based on the MIs, we performed an in silico knockout analysis [36] and depicted the results in a knockout matrix. The knockout matrix in Figure 6 shows the influence of the invasion of antigen (transition: IN_AG_TIS), the production of B cells in the blood (transition: IN_B_BL), the replication of activated B cells in the dark zone (transition: M_B1_DZ), the replication of memory B cells in the medulla (transition: M_B3_ME), the production of antibody by activated B cells (transition: REL_AB_TIS) for the production of antibodies (place: AB_TIS), antigen (place: AG_TIS), and activated B cells with antigen in the light zone (place: B1_AG_LZ). The half-filled circle is colored red if the production of the corresponding species is no longer possible and green otherwise. Trivially, a stop of the invasion of antigen, i.e., a knockout of transition IN_AG_TIS, has the highest impact; antigen in the tissue (place: AG_TIS) and activated B cells with antigen (place: B1_AG_LZ) are no longer produced. Antibody (place: AB_TIS) production is, however, possible without antigens. A stop of the production of B cells, i.e., a knockout of transition IN_B_BL, forbids the production of newly activated B cells (place: B1_AG_LZ). A production of antibodies is, however, possible because of the replication of activated B cells and memory B cells in the dark zone and the medulla. A stop of the replication of activated B cells and memory B cells in the dark zone and the medulla, however, has no effect because each can compensate for the loss of the other. The only way to completely disable an immune response with antibodies would be a knockout of the production of antigens by activated B cells. The insensitivity of the antibody production to single knockouts of either antigen invasion, replication of activated B cells, or replication of memory B cells demonstrates the robustness of the immune response.

### 3.5. Prediction of System Behavior via Simulation

**Figure 7:**
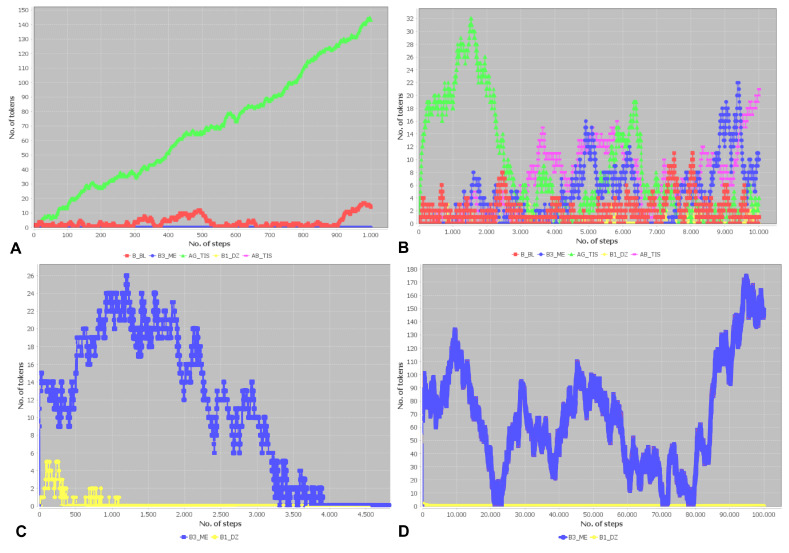
Token flows for various initial physiological conditions. The figures depict the number of antigens (green dots), memory B cells (blue dots), B cells in the blood (red dots), activated B cells (yellow dots), and antibodies (pink dots). (**A**): Initial condition without cells. The antigens accumulate monotonically. No memory B cells are produced, and no immune response is triggered. (**B**): Initial condition with 100 T cells, 100 dendritic cells, 100 antigen-presenting cells, and 100 macrophages. The antigens accumulate only in the initial phase. The system adapts to the antigen. After around 1500 steps, the number of antibodies increases and the number of antigens drops. An adaptive immune response takes place. (**C**): The initial condition is an adapted system (10,000 steps) with no further inflow of antigens. After around 3500 steps, the number of memory B cells drops again to zero. The system loses its memory of the antigen. (**D**): The initial condition is an adapted system (20,000 steps) with no further inflow of antigens. A high number of memory B cells makes the system robust against memory loss. Even after 100,000 steps, the number of memory B cells remains nonzero. Eventually, the number of memory B cells randomly drops to zero and the system loses its memory to the antigen.

To predict the system’s behavior in a nondeterministic way, we performed simulations of the token flow in the PN by applying an asynchronous firing rule. Figure 7A plots the time development of the tokens for the empty initial marking. Figure 7A plots the dynamics of antigens in the tissue (place: AG_TIS, green dots), B cells in the blood (place: B_BL, red dots), activated B cells in the dark zone (place: B1_DZ, yellow dots), memory B cells in the medulla (place: B3_ME, blue dots), and antibodies in the tissue, (place: AB_TIS, pink dots). Whereas the number of antigens increases linearly, the number of B cells in the blood fluctuates on a low level. Neither activated B cells in the dark zone nor memory B cells in the medulla are generated. There is no production of antibodies, and the system shows no immune response.

The lack of tokens on the four PIs hampers the functionality of the system; see Table A4 in the Appendix A. We started the simulation with 100 tokens on each of the places for antigen (AG_TIS), macrophages (M_TIS), antigen-presenting cells (APC_TZ), T cells (T_BL), and dendritic cells (DC_GC). Figure 7B plots the time development of the tokens for an initial marking. The simulation ran for 10,000 steps in an asynchronous simulation mode. The number of antigens (green dots) initially increases. After roughly 1500 steps, the systems adapts to the antigens. Activated B cells in the dark zone (yellow dots) and memory B cells in the medulla (blue dots) are produced, leading to an increase in the number of antibodies (pink dots). The number of antigens drops and fluctuates at a low level. The drop in antigen is a consequence of the increasing production of antibodies by activated B cells. The number of B cells in the blood (red dots) randomly fluctuates at a low level.

We saved the marking after 10,000 steps. We restarted the simulation with the saved marking, now without any inflow of antigens. Figure 7C shows the time development of the number of activated B cells in the dark zone (yellow dots) and memory B cells (blue dots). All activated B cells in the dark zone were lost after 1000 steps and were no longer able to reproduce. While the memory B cells persisted for a far higher number of simulation steps, they were eventually lost after around 4000 simulation steps. After 4000 simulation steps, the system had lost all activated B cells, and would have to adapt from scratch to a possible inflow of antigen. This simulation shows the memory loss of the immune response when not stimulated by the inflow of antigens.

We repeated the in silico experiments for a longer stimulation of the system by antigens. To this end, we saved the marking of the PN after 20,000 simulation steps with an inflow of antigens. Restarting the simulation with the saved marking and without an inflow of antigen yielded the time development in Figure 7D. Whereas the activated B cells in the dark zone (yellow dots) vanished after a few simulation steps, the number of memory B cells was nonzero for the entire simulation of 100,000 steps. Note that the number of memory B cells reaches low numbers several timesat around 20,000, 70,000, and 80,000 simulation steps. If the number of memory B cells had become zero at one of these simulation steps, the memory B cells would have been lost. The number of memory B cells is a result of replication and degradation. The random fluctuation induced by these two counteracting processes results in a certain non-zero probability that the number of memory B cells may reach zero. Without the simulation providing an inflow of antigens, the system eventually loses all memory B cells. After the loss of memory B cells, the system has to re-adapt to any new possible inflow of antigens. This simulation describes the finite time period of immunity to an antigen after an infection or vaccination.

## 4. Discussion and Conclusions

To predict the behavior of the immune system in the lymph node, we constructed a semi-quantitative PN model of the immune system. The components of the model were the major cellular players of the immune response. The advantage of the PN approach is an abstraction level that enables model construction, simulation, and verification even if knowledge of kinetic data, i.e., of numbers for reaction constants or the number of cells, are lacking. Our objective target was the development of a consistent model of the interplay of cells. To validate the consistency of the network, we applied a variety of theoretical methods and algorithms based on invariant properties.

Verification techniques based on invariant properties are well established for PN models of metabolic systems [68] and signal transduction [32,69]. Our model described motile cells that migrate between compartments of the lymph node, interact, replicate, and become activated. To the best of our knowledge, this is the first time that methods of theoretical verification based on invariant properties have been used and evaluated for a model of the concerted action of cells in the lymph node.

We proposed an iterative process of model construction and steps of verification to develop a consistent model. Our approach involved the incorporation of known biological processes and the analysis of invariant properties. We chose the abstraction level of the model with two objectives: first, to make the model as small as possible while including all of the processes that the expert medical pathologists in our group considered essential; second, from the theoretical side, we required that the network be CTI and that each transition invariant be assigned to a biologically meaningful process. Both of these conditions are well-known prerequisites of PN models of metabolic or signal transduction systems [26,32,63,64]. The CTI property indicates a complete and error-free model under the assumption of a system at steady state.

Additionally, we required that the token flow of the model fulfill biologically-inspired conservation properties. Note that a complex model structure may unintentionally describe a non-biological leakage or source of cells. Thus, each addition or removal of a species had to be explicitly modeled by either input/output reactions or replication cycles. To check the conservation properties of cells, we found it worthwhile to compute the PIs not of the network itself, but of the PBOPN. In the PBOPN, we deleted all input and output transitions and set the weights of all arcs to one. By setting all weights to one, we suppressed the replication of cells in our model. Note that other models may use other motifs to describe replication, and as such may require a different strategy to suppress replications. For both the ordinary and PBOPN cases, each place invariant had to correspond to the conservation of a specific cell type, e.g., B cells, macrophages, and T cells. The PBOPN had ten place invariants. In our model, we found one place, representing antibodies, that was not covered by a place invariant. The production of antibodies by activated plasma B cells was the biological justification for this non-covered place.

By applying the iterative process of model construction and verification of invariant properties, we developed our PN model of the lymph node. The final PN had 49 places, 65 transitions, and 149 arcs. The model was covered by 25 TIs and had four PIs. The four PIs represented the conservation of macrophages, T cells, dendritic cells, and antigen-presenting cells. The PBOPN had six additional place invariants representing B cells, activated B cells, and antigens. Note that the inflow of B cells and antigens was explicitly modeled by input transitions in the model. Activated B cells were produced by replication cycles.

The 25 TIs represented the diversity of functional modules. We assigned a specific biological function to each of the 25 TIs. However, we found essential couplings that were not reflected by TIs, e.g., no TI described coupling of the invasion of antigens to the activation of B cells, the production of antibodies, or the replication of activated B cells in the medulla. The shortcomings of TIs to describe complete pathways have been observed for PN models of signal transduction, motivating the concept of Manatee invariants [35]. Our PN model had 66 MIs. Among the 66 MIs, 14 MIs described the production of antibodies. The diversity of MIs describe the functional modules of the immune response in a holistic view, and include essential couplings, e.g., the coupling of antibody invasion to the production of antigen. Through an in silico knockout analysis, we found the production of antibodies to be quite robust against perturbations, such as the replication of activated B cells or the replication of memory B cells. A failure of persistent defense by the immune system would be lethal for an organism; hence, we expect any theoretical model of the immune system to be robust against perturbation.

We applied only known biological reactions. A list of the resources is provided in Table A2 in the Appendix A. To validate the combination of all reactions, we performed simulations. The simulation of our PN model predicted the dynamic behavior expected for an immune response. The systems adapted to infection by antigen and became able to control the number of antigens. The adapted model responded much faster to a second infection and was able to keep the number of antibodies at a low level. The effectiveness of the defense depended on the time period of the infection and on the time period between the first and second infections. After a long time period of the first infection, the defense mechanism became quite stable, though it eventually lost its memory to the antibody. After the loss of its memory, the model had to adapt anew in order to establish an effective immune response. The loss of memory was a random process that varied broadly from simulation to simulation. This behavior of the immune response is known from observations of the duration of the immune defense after infection or vaccination [70,71].

The ability of a PN model to mimic and predict the dynamic behavior of the immune response was surprising, as the model applied neither any quantitative rate constants nor any mass action kinetics. All reactions in the PN model had identical “time scales”. No reaction was faster than another. We assumed that the majority of reactions of our model had similar rates and that the combinatorial diversity of pathways was the determining factor of the dynamic behavior.

Real-time imaging microscopy on human lymph nodes has recorded the velocities and 3D tracks of individual cells [72], while automated image analysis is able to measure the distribution of dimension of lymphatic compartments and the abundances of cells in the departments [57]. We plan to feed quantitative data into our PN model in order to develop a quantitative model. Eventually, we want to develop a model based on ordinary differential equations, stochastic simulation with the Gillespie algorithm, and an agent-based model.

In several aspects, this paper creates a unique basis for further investigations, especially clinical, pharmacological, and machine learning studies. The model presented here can be widened and adopted for the input of quantitative and qualitative human data. These strategies may enable us to understand deeper processes in human lymph nodes, as well as to predict complications and outcomes concerning CAR and NK cell therapies. In addition, despite the complexity of the system, the modulation of the cellular pathways in the model could elucidate aspects of cell interaction rules. Even without following primer causative suggestions, the results drawn from the input data may help in therapeutic decisions.

## Figures and Tables

**Table 1:** Abbreviations for the compartments of the lymph node.

Abbreviation	Name
BL	blood
DZ	dark zone
GC	germinal center
LN	lymph node
LZ	light zone
ME	medulla
SCS	subcapsular sinus
TIS	tissue
TZ	T zone (paracortex)

**Table 2:** Abbreviations for cells and antigen.

Abbreviation	Name	Compartment
AB	antibody	TIS
AG	antigen	BL, TIS
APC	antigen-presenting cells	TZ
B	B cell	BL, TZ, GC, LZ
B1	B cell (activated)	LZ, DZ, GC
B2	B cell (plasma cell)	GC, TZ, ME, BL, TIS
B3	B cell (memory cell)	ME
DC	dendritic cells	GC, LZ
M	macrophages	DZ, GC, LZ, SCS, TIS, TZ
T	T cell	BL, GC, LZ, TZ

**Table 3:** Abbreviations for the types of reactions and processes.

Abbreviation	Name	Description
DIF	differentiation	B cell differentiates to plasma B cell or memory B cell
ENC	encounter	start of interaction between species e.g., cell-cell communication, recognition of antigen
G1	replication	G1 phase of cell replication
IN	influx	interface to the environment that feeds species to the system
M	replication	M phase of cell replication
OUT	out-flux	interface to the environment that takes species from the system, also cell death
REG	regeneration	a cell recovers and becomes active again
REL	release	final step of the transport of an antibody by a B cell
SEP	separation	end of interaction between species as, e.g., cell-cell communication, recognition of antigen
TRA	translocation	species moves from one compartment to another

**Figure A1:**
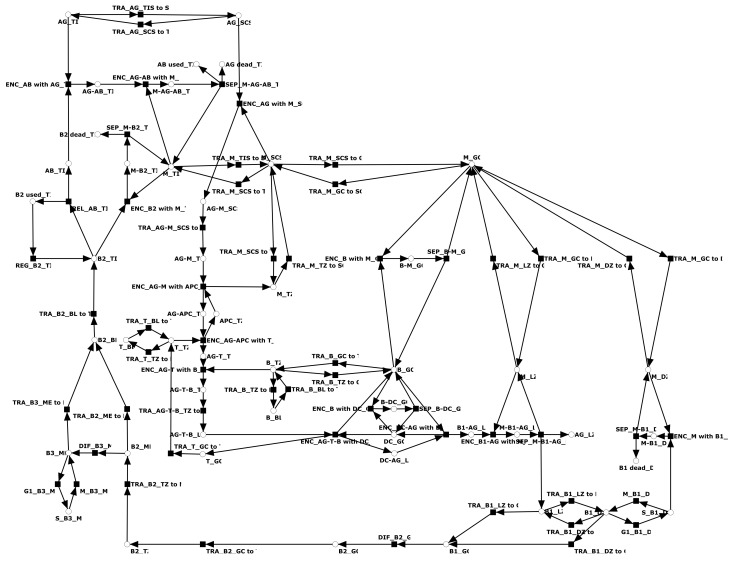
Graphical representation of the PBOPN (see text). The weights of all edges are set to one. All input transitions and all output transitions are deleted.

## Data Availability

Not applicable.

## Supplementary Materials

The following supporting information can be downloaded at: https://www.mdpi.com/article/10.3390/biomedicines11020452/s1, Data S1: Petri net in the format of Systems Biology Markup Language (SBML); Data S2: Petri net in the format of Systems Biology Markup Language, place-bordered version (SBML); Data S3: 66 Manatee invariants (Excel).

## Appendix A

**Table A1:** The 49 places, their names, and a description of their biological meaning. The prefix of the name encodes the species, e.g., a cell, a set of interacting cells, or an antigen. The prefix is either a single abbreviation or a concatenation of abbreviations; for the abbreviations of types of species, refer to Table 2. The suffix of the name encodes the compartment in which the species is located; for the abbreviations of the compartments, refer to Table 1.

No.	Name	Description
P-0	T_BL	T cells in blood
P-1	B_BL	B cells come from blood in the lymph node
P-2	B_TZ	B cells in T zone
P-3	T_TZ	T cells in T zone
P-4	B3_ME	memory B cell in the medulla
P-5	B_GC	B cells in the germinal center
P-6	S_B3_ME	technical place for the replication of memory B cells
P-7	M_GC	macrophages in germinal center
P-8	DC_GC	dendritic cells in germinal center
P-9	B_DC_GC	B cell search for an antigen on dendritic cells in germinal center
P-10	AG_LZ	antigen in light zone before outflow
P-11	M_LZ	macrophages in light zone of germinal center
P-12	M_TZ	macrophages in T zone
P-13	M_B1_AG_LZ	macrophages bind to activated B cells and antigen before antigen will be degraded in the light zone of germinal center
P-14	B1_LZ	activated B cells in light zone
P-15	S_B1_DZ	technical place for the replication of activated B cells
P-16	B_M_GC	B cell and macrophage in germinal center
P-17	APC_TZ	standard localization of antigen-presenting cells in T zone
P-18	M_DZ	macrophages in dark zone of germinal center
P-19	M_B1_DZ	macrophages bind to activated B cells before degradation in the dark zone of germinal center
P-20	M_SCS	macrophages in subcapsular sinus
P-21	B1_dead_DZ	deactivated B cells before degradation in dark zone
P-22	M_B2_TIS	macrophages bind to plasma B cells in tissue
P-23	B2_dead_TIS	deactivated plasma B cell in the tissue
P-24	AG_TIS	antigen will be produced in tissue
P-25	AG_SCS	antigen comes in the lymph node (subcapsular sinus)
P-26	B1_GC	activated B cell in the germinal center
P-27	AG_M_SCS	macrophages catch the antigen in the subcapsular sinus
P-28	AG_M_TZ	macrophages bring the antigen in the T zone
P-29	AG_APC_TZ	antigen-presenting cells show antigen in T zone
P-30	AG_T_TZ	T cells will be activated by antigen in T zone
P-31	AG_T_B_TZ	activated T cells stimulate B cells in T zone
P-32	AG_T_B_LZ	activated T cells and B cells move to the light zone
P-33	DC_AG_LZ	dendritic cell presenting antigen in germinal center
P-34	B1_AG_LZ	activated B cell with antigen in the light zone
P-35	T_GC	T cells in germinal center
P-36	B1_DZ	activated B cells in the dark zone
P-37	B2_GC	plasma B cell in the germinal center
P-38	B2_TZ	plasma B cell in the T zone
P-39	B2_ME	plasma B cell in the medulla
P-40	B2_BL	plasma B cell in the blood
P-41	B2_TIS	plasma B cell in the tissue
P-42	AB_TIS	antibody, freshly produced by a plasma B cell
P-43	B2_used_TIS	plasma B cell before regeneration in the tissue
P-44	AG_AB_TIS	antibody marked antigen for degradation in tissue
P-45	M_TIS	macrophages in tissue
P-46	M_AG_AB_TIS	macrophages bind to the complex of antigen and antibody in tissue
P-47	AG_dead_TIS	antigen will be degraded in tissue
P-48	AB_used_TIS	antibody, inactivated and marked for degradation (technical place)

**Table A2:** List of 65 transitions, their names, and a description of their biological representatives. The prefix of the name encodes the reactions and processes. The abbreviations are explained in Table 3. In the middle are the interacting cells in the abbreviation from Table 2. The suffixes of the names encode the compartment in which the species is located; for the abbreviations of the compartments, refer to Table 1.

No.	Name	Description
T-0	OUT_AG_TIS	Degradation of antigens in the body (tissue) [73]
T-1	OUT_AB_TIS	Degradation of antibodies in the body (tissue) [74]
T-2	TRA_B_BL_to_TZ	Migration of B cells from blood to T zone [75]
T-3	TRA_T_BL_to_TZ	Migration of T cells from blood to T zone [76,77]
T-4	ENC_B2_with_M_TIS	Plasma B cells contact macrophages in tissue [78]
T-5	DIF_B3_ME	Plasma B cells differentiate in memory B cells in medulla [79]
T-6	TRA_B_TZ_to_GC	Migration of B cells from T zone to germinal center [78]
T-7	TRA_T_TZ_to_BL	Migration of T cells from T zone to blood [77]
T-8	M_B3_ME	Technical transition for the replication of memory B cells in medulla [80]
T-9	TRA_B_GC_to_TZ	Migration of B cells from germinal center to T zone [81]
T-10	G1_B3_ME	Technical transition for the replication of memory B cells in medulla [80]
T-11	TRA_B_TZ_to_BL	Migration of B cells from T zone to blood [75]
T-12	TRA_B3_ME_to_BL	Migration of memory B cells from medulla to blood [75]
T-13	TRA_AG_SCS_to_TIS	Migration of antigen from subcapsular sinus in tissue [82]
T-14	IN_B_BL	Production of B cells in bone marrow come in the blood [75]
T-15	SEP_B_DC_GC	B cells search on dendritic cells for antigens after this go apart again [81,83]
T-16	ENC_B_with_DC_GC	B cells contact dendritic cells in germinal center [81]
T-17	TRA_M_GC_to_LZ	Migration of macrophages from the unspecific germinal center in the light zone [83]
T-18	ENC_B1_AG_with_M_LZ	Activated B cells with antigen contact macrophages in the light zone of germinal center [83]
T-19	SEP_M_B1_AG_LZ	Macrophages mark antigens for degradation after the activation of B cells in the light zone of germinal center [83]
T-20	TRA_M_LZ_to_GC	Migration of macrophages from light zone to the unspecific germinal center
T-21	OUT_AG_LZ	Technical transition for the degradation of antigens in the light zone of the germinal center
T-22	M_B1_DZ	Technical transition for the replication of activated B cells in the dark zone of germinal center [84]
T-23	ENC_B_with_M_GC	B cells contact macrophages in germinal center [84]
T-24	G1_B1_DZ	Technical transition for the replication of activated B cells in the dark zone of germinal center [85]
T-25	SEP_B_M_GC	Macrophages search for incorrect B cells in the germinal center, after this, go apart again [84]
T-26	IN_AG_TIS	Influx of antigen, like viruses or bacteria, in the body (tissue) [86,87,88]
T-27	OUT_B_BL	Technical transition for the degradation of B cells in blood
T-28	TRA_M_GC_to_DZ	Migration of macrophages from the unspecific germinal center in dark zone [83]
T-29	TRA_M_DZ_to_GC	Migration of macrophages from dark zone to the unspecific germinal center [83]
T-30	ENC_M_with_B1_DZ	Activated B cells contact macrophages in the dark zone of germinal center [83]
T-31	SEP_M_B1_DZ	Activated B cells will mark by macrophages for degradation in the dark zone of the germinal center [88]
T-32	TRA_M_SCS_to_TZ	Migration of macrophages from subcapsular sinus to T zone [89]
T-33	OUT_B1_dead_DZ	Degradation of non-specialized activated B cells in the dark zone of germinal center [83,90]
T-34	TRA_M_TZ_to_SCS	Migration of macrophages from T zone to subcapsular sinus [89]
T-35	TRA_M_SCS_to_GC	Migration of macrophages from subcapsular sinus to the unspecific germinal center [89,91]
T-36	SEP_M_B2_TIS	Plasma B cells will mark by macrophages for degradation in the body (tissue) [92]
T-37	TRA_M_GC_to_SCS	Migration of macrophages from the unspecific germinal center to subcapsular sinus [83]
T-38	OUT_B2_TIS	Degradation of plasma B cells after the release of antibodies in the body (tissue) [79]
T-39	TRA_AG_TIS_to_SCS	Migration of antigen from tissue to subcapsular sinus [82]
T-40	ENC_AG_with_M_SCS	Macrophages contact antigens in subcapsular sinus [89,93]
T-41	TRA_B1_LZ_to_GC	Migration of activated B cells from the light zone in the unspecific germinal center [83]
T-42	TRA_AG_M_SCS_to_TZ	Migration of activated macrophages by antigen from subcapsular sinus in T zone [89]
T-43	TRA_B1_DZ_to_GC	Migration of activated B cells from the dark zone in the unspecific germinal center [83]
T-44	ENC_AG_M_with_APC_TZ	Antigen-presenting cells contact the antigen and macrophages in T zone [94]
T-45	ENC_AG_APC_with_T_TZ	T cells contact antigen on antigen-presenting cells in T zone [95,96]
T-46	ENC_AG_T_with_B_TZ	B cells contact the complex of antigen and T cell in T zone [96,97]
T-47	TRA_AG_T_B_TZ_to_LZ	Migration of activated T and B cells by antigen from T zone to light zone of germinal center [79,97]
T-48	ENC_AG_T_B_with_DC_LZ	Dendritic cells contact the complex of antigen, T and B cell in the light zone of germinal center [76]
T-49	ENC_DC_AG_with_B_LZ	B cells contact dendritic cells with antigen in the light zone of germinal center [81,88]
T-50	TRA_T_GC_to_TZ	Migration of T cells from the unspecific germinal center to T zone [98]
T-51	TRA_B1_LZ_to_DZ	Migration of activated B cells from light to dark zone of germinal center [99]
T-52	DIF_B2_GC	Activated B cells differentiate to plasma B cells in germinal center [100]
T-53	TRA_B2_GC_to_TZ	Migration of plasma B cells from germinal center to T zone [83]
T-54	TRA_B2_TZ_to_ME	Migration of plasma B cells from T zone to medulla [101]
T-55	TRA_B2_ME_to_BL	Migration of plasma B cells from medulla to blood [75]
T-56	TRA_B1_DZ_to_LZ	Migration of activated B cells from dark to the light zone of the germinal center [83,99,102]
T-57	TRA_B2_BL_to_TIS	Migration of plasma B cells from blood to the body (tissue) [75]
T-58	REL_AB_TIS	Release of antibodies by plasma B cells in the body (tissue) [82]
T-59	REG_B2_TIS	Regeneration of plasma B cells after the release of antibodies before plasma B cells can release antibodies again [82,101]
T-60	ENC_AB_with_AG_TIS	Antibodies contact antigens in tissue [103]
T-61	TRA_M_TIS_to_SCS	Migration of macrophages from the body (tissue) to subcapsular sinus [89]
T-62	TRA_M_SCS_to_TIS	Migration of macrophages from subcapsular sinus in the body (tissue) [89]
T-63	ENC_AG_AB_with_M_TIS	Macrophages contact the complex of antigen and antibody in tissue [92]
T-64	SEP_M_AG_AB_TIS	Antigens and antibodies will be marked by macrophages for degradation in the body (tissue) [92]

**Table A3:** Reaction system. The species “OExternal” denotes the interface to the environment. “OExternal” is ignored for preconditions and token balance.

No.	Name	Reaction
0 :	OUT_AG_TIS :	AG_dead_TIS → OExternal .
1 :	OUT_AB_TIS :	AB_used_TIS → OExternal .
2 :	TRA_B_BL_to_TZ :	B_BL → B_TZ .
3 :	TRA_T_BL_to_TZ :	T_BL → T_TZ .
4 :	ENC_B2_with_M_TIS :	B2_TIS + M_TIS → M_B2_TIS .
5 :	DIF_B3_ME :	B2_ME → B3_ME .
6 :	TRA_B_TZ_to_GC :	B_TZ → B_GC .
7 :	TRA_T_TZ_to_BL :	T_TZ → T_BL .
8 :	M_B3_ME :	S_B3_ME → 2 B3_ME .
9 :	TRA_B_GC_to_TZ :	B_GC → B_TZ .
10 :	G1_B3_ME :	B3_ME → S_B3_ME .
11 :	TRA_B_TZ_to_BL :	B_TZ → B_BL .
12 :	TRA_B3_ME_to_BL :	B3_ME → B2_BL .
13 :	TRA_AG_SCS_to_TIS :	AG_SCS → AG_TIS .
14 :	IN_B_BL :	OExternal → B_BL .
15 :	SEP_B_DC_GC :	B_DC_GC → B_GC + DC_GC .
16 :	ENC_B_with_DC_GC :	B_GC + DC_GC → B_DC_GC .
17 :	TRA_M_GC_to_LZ :	M_GC → M_LZ .
18 :	ENC_B1_AG_with_M_LZ :	M_LZ + B1_AG_LZ → M_B1_AG_LZ .
19 :	SEP_M_B1_AG_LZ :	M_B1_AG_LZ → AG_LZ + M_LZ + B1_LZ .
20 :	TRA_M_LZ_to_GC :	M_LZ → M_GC .
21 :	OUT_AG_LZ :	AG_LZ → OExternal .
22 :	M_B1_DZ :	S_B1_DZ → 2 B2_DZ .
23 :	ENC_B_with_M_GC :	B_GC + M_GC → B_M_GC .
24 :	G1_B1_DZ :	B2_DZ → S_B1_DZ .
25 :	SEP_B_M_GC :	B_M_GC → B_GC + M_GC .
26 :	IN_AG_TIS :	OExternal → AG_TIS .
27 :	OUT_B_BL :	B_BL → OExternal .
28 :	TRA_M_GC_to_DZ :	M_GC → M_DZ .
29 :	TRA_M_DZ_to_GC :	M_DZ → M_GC .
30 :	ENC_M_with_B1_DZ :	2 S_B1_DZ + M_DZ → M_B1_DZ .
31 :	SEP_M_B1_DZ :	M_B1_DZ → M_DZ + B1_dead_DZ .
32 :	TRA_M_SCS_to_TZ :	M_SCS → M_TZ .
33 :	OUT_B1_dead_DZ :	B1_dead_DZ → OExternal .
34 :	TRA_M_TZ_to_SCS :	M_TZ → M_SCS .
35 :	TRA_M_SCS_to_GC :	M_SCS → M_GC .
36 :	SEP_M_B2_TIS :	M_B2_TIS → B2_dead_TIS + M_TIS .
37 :	TRA_M_GC_to_SCS :	M_GC → M_SCS .
38 :	OUT_B2_TIS :	B2_dead_TIS → OExternal .
39 :	TRA_AG_TIS_to_SCS :	AG_TIS → AG_SCS .
40 :	ENC_AG_with_M_SCS :	M_SCS + AG_SCS → AG_M_SCS .
41 :	TRA_B1_LZ_to_GC :	B1_LZ → B1_GC .
42 :	TRA_AG_M_SCS_to_TZ :	AG_M_SCS → AG_M_TZ .
43 :	TRA_B1_DZ_to_GC :	B2_DZ → B1_GC .
44 :	ENC_AG_M_with_APC_TZ :	APC_TZ + AG_M_TZ →
		M_TZ + AG_APC_TZ .
45 :	ENC_AG_APC_with_T_TZ :	T_TZ + AG_APC_TZ →
		APC_TZ + AG_T_TZ .
46 :	ENC_AG_T_with_B_TZ :	B_TZ + AG_T_TZ → AG_T_B_TZ .
47 :	TRA_AG_T_B_TZ_to_LZ :	AG_T_B_TZ → AG_T_B_LZ .
48 :	ENC_AG_T_B_with_DC_LZ :	DC_GC + AG_T_B_LZ →
		DC_GC + B1_AG_LZ .
50 :	TRA_T_GC_to_TZ :	T_GC → T_TZ .
51 :	TRA_B1_LZ_to_DZ :	B1_LZ → B2_DZ .
52 :	DIF_B2_GC :	B1_GC → B2_GC .
53 :	TRA_B2_GC_to_TZ :	B2_GC → B2_TZ .
54 :	TRA_B2_TZ_to_ME :	B2_TZ → B2_ME .
55 :	TRA_B2_ME_to_BL :	B2_ME → B2_BL .
56 :	TRA_B1_DZ_to_LZ :	B2_DZ → B1_LZ .
57 :	TRA_B2_BL_to_TIS :	B2_BL → B2_TIS .
58 :	REL_AB_TIS :	B2_TIS → AB_TIS + B2_used_TIS .
59 :	REG_B2_TIS :	B2_used_TIS → B2_TIS .
60 :	ENC_AB_with_AG_TIS :	AG_TIS + AB_TIS → AG_AB_TIS .
61 :	TRA_M_TIS_to_SCS :	M_TIS → M_SCS .
62 :	TRA_M_SCS_to_TIS :	M_SCS → M_TIS .
63 :	ENC_AG_AB_with_M_TIS :	AG_AB_TIS + M_TIS → M_AG_AB_TIS .
64 :	SEP_M_AG_AB_TIS :	M_AG_AB_TIS →
		M_TIS + AG_dead_TIS + AB_used_TIS .

**Table A4:** List of four place invariants (PIs). The columns ”length”, ”places”, and ”conserved cell” provide the number of places of the PI, the names of all places of the PI, and the conservation of which cell (type) the PI originates from, respectively.

No.	Length	Places	Conserved Cell
PI-0	13	M_GC, M_LZ, M_TZ, M_B1_AG_LZ, B_M_GC, M_DZ, M_B1_DZ, M_SCS, M_B2_TIS, AG_M_SCS, AG_M_TZ, M_TIS, M_AG_AB_TIS	Macrophages (M)
PI-1	6	T_BL, T_TZ, AG_T_TZ, AG_T_B_TZ, AG_T_B_LZ, T_GC	T cell (T)
PI-2	3	DC_GC, B_DC_GC, DC_AG_LZ	Dendritic cell (DC)
PI-3	2	APC_TZ, AG_APC_TZ	Antigen-presenting cell (APC)

**Table A5:** List of 25 transition invariants (TIs). The columns ”length”, ”transitions”, and ”function” provide the number of transitions of the TI, the names of all transitions of the TI, and a description of the biological meaning of the TI, respectively.

No.	Length	Transitions	Function
TI-0	26	TRA_B_BL_to_TZ ENC_B2_with_M_TIS IN_B_BL ENC_B1_AG_with_M_LZ SEP_M_B1_AG_LZ OUT_AG_LZ IN_AG_TIS TRA_M_TZ_to_SCS SEP_M_B2_TIS OUT_B2_TIS TRA_AG_TIS_to_SCS ENC_AG_with_M_SCS TRA_B1_LZ_to_GC TRA_AG_M_SCS_to_TZ ENC_AG_M_with_APC_TZ ENC_AG_APC_with_T_TZ ENC_AG_T_with_B_TZ TRA_AG_T_B_TZ_to_LZ ENC_AG_T_B_with_DC_LZ ENC_DC_AG_with_B_LZ TRA_T_GC_to_TZ DIF_B2_GC TRA_B2_GC_to_TZ TRA_B2_TZ_to_ME TRA_B2_ME_to_BL TRA_B2_BL_to_TIS	Activation of a B cell by an invading antigen. The activated B cell moves from blood to the T zone and via the light zone, dark zone, medulla, and blood to the tissue. The B cell differentiates in several steps and eventually becomes degraded in the tissue. Neither a cell replication nor the production of any antibody is involved.
TI-1	2	TRA_M_TIS_to_SCS TRA_M_SCS_to_TIS	Macrophages migrate from tissue in SCS and vice versa.
TI-2	2	IN_B_BL OUT_B_BL	Flow of B cells in the blood.
TI-3	2	TRA_T_BL_to_TZ TRA_T_TZ_to_BL	T cells migrate from blood to T zone and vice versa.
TI-4	7	ENC_B2_with_M_TIS M_B3_ME G1_B3_ME TRA_B3_ME_to_BL SEP_M_B2_TIS OUT_B2_TIS TRA_B2_BL_to_TIS	Memory B cells (B3) proliferate in the medulla. Plasma B cells (B2) of in the tissue and are degraded.
TI-5	22	2*TRA_B_BL_to_TZ 2*IN_B_BL 2*ENC_B1_AG_with_M_LZ 2*SEP_M_B1_AG_LZ 2*OUT_AG_LZ 2*G1_B1_DZ 2*IN_AG_TIS ENC_M_with_B1_DZ SEP_M_B1_DZ OUT_B1_dead_DZ 2*TRA_M_TZ_to_SCS 2*TRA_AG_TIS_to_SCS 2*ENC_AG_with_M_SCS 2*TRA_AG_M_SCS_to_TZ 2*ENC_AG_M_with_APC_TZ 2*ENC_AG_APC_with_T_TZ 2*ENC_AG_T_with_B_TZ 2*TRA_AG_T_B_TZ_to_LZ 2*ENC_AG_T_B_with_DC_LZ 2*ENC_DC_AG_with_B_LZ 2*TRA_T_GC_to_TZ 2*TRA_B1_LZ_to_DZ	Activation of a B cell by an invading antigen. The activated B cell (B1) will be degraded in the DZ.
TI-6	2	TRA_B_TZ_to_GC TRA_B_GC_to_TZ	B cells migrate from the T zone to GC and vice versa.
TI-7	2	TRA_M_SCS_to_GC TRA_M_GC_to_SCS	Macrophages migrate from SCS to GC and vice versa.
TI-8	28	TRA_B_BL_to_TZ ENC_B2_with_M_TIS DIF_B3_ME TRA_B3_ME_to_BL IN_B_BL ENC_B1_AG_with_M_LZ SEP_M_B1_AG_LZ OUT_AG_LZ IN_AG_TIS TRA_M_TZ_to_SCS SEP_M_B2_TIS OUT_B2_TIS TRA_AG_TIS_to_SCS ENC_AG_with_M_SCS TRA_AG_M_SCS_to_TZ TRA_B1_DZ_to_GC ENC_AG_M_with_APC_TZ ENC_AG_APC_with_T_TZ ENC_AG_T_with_B_TZ TRA_AG_T_B_TZ_to_LZ ENC_AG_T_B_with_DC_LZ ENC_DC_AG_with_B_LZ TRA_T_GC_to_TZ TRA_B1_LZ_to_DZ DIF_B2_GC TRA_B2_GC_to_TZ TRA_B2_TZ_to_ME TRA_B2_BL_to_TIS	Activation of a B cell by an invading antigen. The activated B cell moves from blood to the T zone and via the light zone, dark zone, medulla, and blood to the tissue. The B cell differentiates in several steps and eventually becomes degraded in the tissue. Neither a cell replication nor the production of any antibody is involved.
TI-9	8	OUT_AG_TIS OUT_AB_TIS IN_AG_TIS REL_AB_TIS REG_B2_TIS ENC_AB_with_AG_TIS ENC_AG_AB_with_M_TIS SEP_M_AG_AB_TIS	Plasma B cells (B2) release antibodies (AB). AB encounters antigen and are degraded.
TI-10	27	TRA_B_BL_to_TZ ENC_B2_with_M_TIS DIF_B3_ME TRA_B3_ME_to_BL IN_B_BL ENC_B1_AG_with_M_LZ SEP_M_B1_AG_LZ OUT_AG_LZ IN_AG_TIS TRA_M_TZ_to_SCS SEP_M_B2_TIS OUT_B2_TIS TRA_AG_TIS_to_SCS ENC_AG_with_M_SCS TRA_B1_LZ_to_GC TRA_AG_M_SCS_to_TZ ENC_AG_M_with_APC_TZ ENC_AG_APC_with_T_TZ ENC_AG_T_with_B_TZ TRA_AG_T_B_TZ_to_LZ ENC_AG_T_B_with_DC_LZ ENC_DC_AG_with_B_LZ TRA_T_GC_to_TZ DIF_B2_GC TRA_B2_GC_to_TZ TRA_B2_TZ_to_ME TRA_B2_BL_to_TIS	Activation of a B cell by an invading antigen. The activated B cell moves from blood to the T zone and via the light zone, dark zone, medulla, and blood to the tissue. The B cell differentiates in several steps and eventually becomes degraded in the tissue. Neither a cell replication nor the production of any antibody is involved.
TI-11	2	TRA_B_BL_to_TZ TRA_B_TZ_to_BL	B cells migrate from blood to the T zone and vice versa.
TI-12	2	ENC_B_with_M_GC SEP_B_M_GC	B cells encounter macrophages in GC and separate.
TI-13	2	TRA_AG_SCS_to_TIS TRA_AG_TIS_to_SCS	Antigen moves from tissue to SCS and vice versa.
TI-14	2	TRA_M_GC_to_DZ TRA_M_DZ_to_GC	Macrophages migrate from GC to DZ and vice versa.
TI-15	2	SEP_B_DC_GC ENC_B_with_DC_GC	B cells encounter dendritic cells in GC and separate.
TI-16	13	ENC_B2_with_M_TIS DIF_B3_ME TRA_B3_ME_to_BL M_B1_DZ G1_B1_DZ SEP_M_B2_TIS OUT_B2_TIS TRA_B1_LZ_to_GC DIF_B2_GC TRA_B2_GC_to_TZ TRA_B2_TZ_to_ME TRA_B1_DZ_to_LZ TRA_B2_BL_to_TIS	Activated B cells (B1) proliferate in DZ. B1 differentiates to plasma cells (B2) and moves via the light zone of the germinal center, and the T zone in the medulla. In the medulla, B2 differentiates to memory B cells (B3). B2 moves then via blood in the tissue and will there be degraded.
TI-17	2	TRA_M_GC_to_LZ TRA_M_LZ_to_GC	Macrophages migrate from GC to LZ and vice versa.
TI-18	2	TRA_M_SCS_to_TZ TRA_M_TZ_to_SCS	Macrophages migrate from T zone to SCS and vice versa.
TI-19	2	TRA_B1_LZ_to_DZ TRA_B1_DZ_to_LZ	Activated B cells (B1) migrate from LZ to DZ and vice versa.
TI-20	5	2*M_B1_DZ 4*G1_B1_DZ ENC_M_with_B1_DZ SEP_M_B1_DZ OUT_B1_dead_DZ	Activated B cells (B1) proliferate in DZ and is degraded.
TI-21	12	ENC_B2_with_M_TIS M_B1_DZ G1_B1_DZ SEP_M_B2_TIS OUT_B2_TIS TRA_B1_LZ_to_GC DIF_B2_GC TRA_B2_GC_to_TZ TRA_B2_TZ_to_ME TRA_B2_ME_to_BL TRA_B1_DZ_to_LZ TRA_B2_BL_to_TIS	Activated B cells (B1) proliferate in DZ. B1 differentiates to plasma cells (B2) and moves via the light zone of the germinal center, T zone, medulla, and blood in the tissue. B2 is degraded in the tissue.
TI-22	12	ENC_B2_with_M_TIS DIF_B3_ME TRA_B3_ME_to_BL M_B1_DZ G1_B1_DZ SEP_M_B2_TIS OUT_B2_TIS TRA_B1_DZ_to_GC DIF_B2_GC TRA_B2_GC_to_TZ TRA_B2_TZ_to_ME TRA_B2_BL_to_TIS	Activated B cells (B1) proliferate in DZ. B1 differentiates to plasma cells (B2) and moves via germinal center, and T zone in the medulla. In the medulla, B2 differentiates to memory B cells (B3). B2 moves then via blood in the tissue and will there be degraded.
TI-23	27	TRA_B_BL_to_TZ ENC_B2_with_M_TIS IN_B_BL ENC_B1_AG_with_M_LZ SEP_M_B1_AG_LZ OUT_AG_LZ IN_AG_TIS TRA_M_TZ_to_SCS SEP_M_B2_TIS OUT_B2_TIS TRA_AG_TIS_to_SCS ENC_AG_with_M_SCS TRA_AG_M_SCS_to_TZ TRA_B1_DZ_to_GC ENC_AG_M_with_APC_TZ ENC_AG_APC_with_T_TZ ENC_AG_T_with_B_TZ TRA_AG_T_B_TZ_to_LZ ENC_AG_T_B_with_DC_LZ ENC_DC_AG_with_B_LZ TRA_T_GC_to_TZ TRA_B1_LZ_to_DZ DIF_B2_GC TRA_B2_GC_to_TZ TRA_B2_TZ_to_ME TRA_B2_ME_to_BL TRA_B2_BL_to_TIS	Activation of a B cell by an invading antigen. The activated B cell moves from blood to the T zone and via the light zone, dark zone, medulla, and blood to the tissue. The B cell differentiates in several steps and eventually becomes degraded in the tissue. Neither a cell replication nor the production of any antibody is involved.
TI-24	11	ENC_B2_with_M_TIS M_B1_DZ G1_B1_DZ SEP_M_B2_TIS OUT_B2_TIS TRA_B1_DZ_to_GC DIF_B2_GC TRA_B2_GC_to_TZ TRA_B2_TZ_to_ME TRA_B2_ME_to_BL TRA_B2_BL_to_TIS	Activated B cells (B1) proliferate in DZ. B1 differentiates to plasma cells (B2) and moves via germinal center, T zone, medulla, and blood in the tissue. B2 is degraded in the tissue.

**Table A6:** List of the 66 Manatee invariants (MIs). The columns “pure” and “antibody” indicate whether the MI is pure and whether the production of antibodies is part of the MI, respectively. The column “replication” indicates in which compartment(s) replication of activated B cells or memory B cells can occur; ME means the medulla and DZ means the dark zone. MIs that describe no replication of cells have an empty entry in the “replication” column.

No.	No. of Transitions	TI’s	Pure	Antibody	Replication
MI-0	15	TI-4 TI-9		y	ME
MI-1	2	TI-1			
MI-2	2	TI-2	y		
MI-3	2	TI-3			
MI-4	7	TI-4			ME
MI-5	4	TI-2 TI-11	y		
MI-6	24	TI-5 TI-6			DZ
MI-7	2	TI-7			
MI-8	17	TI-4 TI-9 TI-13		y	ME
MI-9	30	TI-0 TI-6 TI-19			
MI-10	36	TI-6 TI-9 TI-23		y	
MI-11	30	TI-6 TI-8			
MI-12	29	TI-6 TI-10			
MI-13	29	TI-6 TI-23			
MI-14	2	TI-14			
MI-15	36	TI-6 TI-9 TI-10		y	
MI-16	13	TI-16			DZ
MI-17	2	TI-17			
MI-18	2	TI-18			
MI-19	6	TI-2 TI-6 TI-11	y		
MI-20	5	TI-20			DZ
MI-21	12	TI-21			DZ
MI-22	12	TI-22			DZ
MI-23	25	TI-5 TI-6 TI-11			DZ
MI-24	11	TI-24			DZ
MI-25	13	TI-19 TI-24			DZ
MI-26	25	TI-5 TI-6 TI-13			DZ
MI-27	14	TI-19 TI-22			DZ
MI-28	13	TI-19 TI-21			DZ
MI-29	28	TI-0 TI-6			
MI-30	7	TI-19 TI-20			DZ
MI-31	14	TI-16 TI-19			DZ
MI-32	25	TI-5 TI-6 TI-19			DZ
MI-33	37	TI-6 TI-8 TI-9		y	
MI-34	31	TI-6 TI-8 TI-11			
MI-35	31	TI-6 TI-8 TI-13			
MI-36	30	TI-6 TI-19 TI-23			
MI-37	35	TI-0 TI-6 TI-9		y	
MI-38	30	TI-6 TI-13 TI-23			
MI-39	29	TI-0 TI-6 TI-11			
MI-40	19	TI-9 TI-24		y	DZ
MI-41	30	TI-6 TI-11 TI-23			
MI-42	29	TI-0 TI-6 TI-13			
MI-43	20	TI-9 TI-22		y	DZ
MI-44	20	TI-9 TI-21		y	DZ
MI-45	21	TI-9 TI-16		y	DZ
MI-46	31	TI-6 TI-10 TI-19			
MI-47	30	TI-6 TI-10 TI-13			
MI-48	30	TI-6 TI-10 TI-11			
MI-49	30	TI-0 TI-6 TI-15			
MI-50	31	TI-6 TI-8 TI-19			
MI-51	31	TI-6 TI-15 TI-23			
MI-52	31	TI-6 TI-10 TI-15			
MI-53	32	TI-6 TI-8 TI-15			
MI-54	26	TI-5 TI-6 TI-12			DZ
MI-55	32	TI-6 TI-8 TI-12			
MI-56	31	TI-6 TI-10 TI-12			
MI-57	26	TI-5 TI-6 TI-15			DZ
MI-58	31	TI-6 TI-12 TI-23			
MI-59	23	TI-9 TI-15 TI-16		y	DZ
MI-60	22	TI-9 TI-13 TI-21		y	DZ
MI-61	22	TI-9 TI-13 TI-22		y	DZ
MI-62	30	TI-0 TI-6 TI-12			
MI-63	21	TI-9 TI-13 TI-24		y	DZ
MI-64	8	TI-2 TI-6 TI-11 TI-12			
MI-65	8	TI-2 TI-6 TI-11 TI-15			

**Table A7:** List of ten place invariants (PIs) of the place-bordered and ordinary Petri net. The columns with “length”, “places”, and “conserved cell” provide the number of places of the PI, the names of all places of the PI, and the conservation of which cell (type) the PI originates from, respectively.

No.	Length	Places	Conserved Cell
PI-0	6	T_BL, T_TZ, AG_T_TZ, AG_T_B_TZ, AG_T_B_LZ, T_GC	T cell (T)
PI-1	2	APC_TZ, AG_APC_TZ	Antigen-presenting cell (APC)
PI-2	3	DC_GC, B_DC_GC, DC_AG_LZ	Dendritic cell (DC)
PI-3	13	M_GC, M_LZ, M_TZ, M_B1_AG_LZ, B_M_GC, M_DZ, M_B1_DZ, M_SCS, M_B2_TIS, AG_M_SCS, AG_M_TZ, M_TIS, M_AG_AB_TIS	Macrophage (M)
PI-4	25	B_BL, B_TZ, B3_ME, B_GC, S_B3_ME, B_DC_GC, M_B1_AG_LZ, B1_LZ, S_B1_DZ, B_M_GC, M_B1_DZ, B1_dead_DZ, M_B2_TIS, B2_dead_TIS, B1_GC, AG_T_B_TZ, AG_T_B_LZ, B1_AG_LZ, B1_DZ, B2_GC, B2_TZ, B2_ME, B2_BL, B2_TIS, B2_used_TIS	B cell (B1, B2, B3)
PI-5	30	B3_ME, S_B3_ME, M_B1_AG_LZ, B1_LZ, S_B1_DZ, M_B1_DZ, B1_dead_DZ, M_B2_TIS, B2_dead_TIS, AG_TIS, AG_SCS, B1_GC, AG_M_SCS, AG_M_TZ, AG_APC_TZ, AG_T_TZ, AG_T_B_TZ, AG_T_B_LZ, DC_AG_LZ, B1_AG_LZ, B1_DZ, B2_GC, B2_TZ, B2_ME, B2_BL, B2_TIS, B2_used_TIS, AG_AB_TIS, M_AG_AB_TIS, AB_used_TIS	Cells activated by antigen (AG).
PI-6	30	B3_ME, S_B3_ME, M_B1_AG_LZ, B1_LZ, S_B1_DZ, M_B1_DZ, B1_dead_DZ, M_B2_TIS, B2_dead_TIS, AG_TIS, AG_SCS, B1_GC, AG_M_SCS, AG_M_TZ, AG_APC_TZ, AG_T_TZ, AG_T_B_TZ, AG_T_B_LZ, DC_AG_LZ, B1_AG_LZ, B1_DZ, B2_GC, B2_TZ, B2_ME, B2_BL, B2_TIS, B2_used_TIS, AG_AB_TIS, M_AG_AB_TIS, AG_dead_TIS	Cells activated by antigen (AG).
PI-7	10	B_BL, B_TZ, B_GC, B_DC_GC, AG_LZ, M_B1_AG_LZ, B_M_GC, AG_T_B_TZ, AG_T_B_LZ, B1_AG_LZ	Non-activated B cell (B) and B cell (B1) with antigen (AG)
PI-8	15	AG_LZ, M_B1_AG_LZ, AG_TIS, AG_SCS, AG_M_SCS, AG_M_TZ, AG_APC_TZ, AG_T_TZ, AG_T_B_TZ, AG_T_B_LZ, DC_AG_LZ, B1_AG_LZ, AG_AB_TIS, M_AG_AB_TIS, AB_used_TIS	Antigen (AG)
PI-9	15	AG_LZ, M_B1_AG_LZ, AG_TIS, AG_SCS, AG_M_SCS, AG_M_TZ, AG_APC_TZ, AG_T_TZ, AG_T_B_TZ, AG_T_B_LZ, DC_AG_LZ, B1_AG_LZ, AG_AB_TIS, M_AG_AB_TIS, AG_dead_TIS	Antigen (AG)

## References

[1] Margaris K., Black R.A. (2012). Modelling the lymphatic system: Challenges and opportunities. J. R. Soc. Interface.

[2] Cappuccio A., Tieri P., Castiglione F. (2016). Multiscale modelling in immunology: A review. Briefings Bioinform..

[3] Eftimie R., Gillard J.J., Cantrell D.A. (2016). Mathematical models for immunology: Current state of the art and future research directions. Bull. Math. Biol..

[4] Moore J.E., Bertram C.D. (2018). Lymphatic system flows. Annu. Rev. Fluid Mech..

[5] Mozokhina A., Savinkov R. (2020). Mathematical modelling of the structure and function of the lymphatic system. Mathematics.

[6] Altan-Bonnet G., Mora T., Walczak A.M. (2020). Quantitative immunology for physicists. Phys. Rep..

[7] Azarov I., Peskov K., Helmlinger G., Kosinsky Y. (2019). Role of T cell-to-dendritic cell chemoattraction in T cell priming initiation in the lymph node: An agent-based modeling study. Front. Immunol..

[8] Keşmir C., De Boer R.J. (1999). A mathematical model on germinal center kinetics and termination. J. Immunol..

[9] Miao H., Hollenbaugh J.A., Zand M.S., Holden-Wiltse J., Mosmann T.R., Perelson A.S., Wu H., Topham D.J. (2010). Quantifying the early immune response and adaptive immune response kinetics in mice infected with Influenza A virus. J. Virol..

[10] Swerdlin N., Cohen I.R., Harel D. (2008). The lymph node B cell immune response: Dynamic analysis in-silico. Proc. IEEE.

[11] Kislitsyn A., Savinkov R., Novkovic M., Onder L., Bocharov G. (2015). Computational approach to 3D modeling of the lymph node geometry. Computation.

[12] Pennisi M., Cavalieri S., Motta S., Pappalardo F. (2016). A methodological approach for using high-level Petri nets to model the immune system response. BMC Bioinform..

[13] Obaid A., Naz A., Ikram A., Awan F.M., Raza A., Ahmad J., Ali A. (2018). Model of the adaptive immune response system against HCV infection reveals potential immunomodulatory agents for combination therapy. Sci. Rep..

[14] Thomas M.J., Klein U., Lygeros J., Rodríguez Martínez M. (2019). A probabilistic model of the germinal center reaction. Front. Immunol..

[15] Pernice S., Follia L., Maglione A., Pennisi M., Pappalardo F., Novelli F., Clerico M., Beccuti M., Cordero F., Rolla S. (2020). Computational modeling of the immune response in multiple sclerosis using epimod framework. BMC Bioinform..

[16] Gutowska K., Formanowicz D., Formanowicz P. (2019). Selected aspects of tobacco-induced prothrombotic state, inflammation and oxidative stress: Modeled and analyzed using petri nets. Interdiscip. Sci. Comput. Life Sci..

[17] Rżosińska K., Formanowicz D., Formanowicz P. (2017). The study of the influence of micro-environmental signals on macrophage differentiation using a quantitative Petri net based model. Arch. Control Sci..

[18] Petri C.A. (1962). Kommunikation mit Automaten. Ph.D. Thesis.

[19] Lipton R. (1976). The Reachability Problem Requires Exponential Space.

[20] Mayr E.W. An algorithm for the general Petri net reachability problem. Proceedings of the Thirteenth Annual ACM Symposium on Theory of Computing. Association for Computing Machinery.

[21] Kostin A.E. (2003). Reachability analysis in T-invariant-less Petri nets. IEEE Trans. Autom. Control.

[22] Kostin A.E. (2006). A reachability algorithm for general Petri nets based on transition invariants. Proceedings of the International Symposium on Mathematical Foundations of Computer Science.

[23] Kostin A. (2008). Using Transition Invariants for Reachability Analysis of Petri Nets.

[24] Leroux J. (2022). The reachability problem for Petri nets is not primitive recursive. Proceedings of the 2021 IEEE 62nd Annual Symposium on Foundations of Computer Science (FOCS).

[25] Czerwiński W., Orlikowski Ł. (2022). Reachability in Vector Addition Systems is Ackermann-Complete. Proceedings of the 2021 IEEE 62nd Annual Symposium on Foundations of Computer Science (FOCS).

[26] Koch I., Reisig W., Schreiber F. (2010). Modeling in Systems Biology: The Petri Net Approach.

[27] Formanowicz D., Wanic-Kossowska M., Pawliczak E., Radom M., Formanowicz P. (2015). Usefulness of serum interleukin-18 in predicting cardiovascular mortality in patients with chronic kidney disease–systems and clinical approach. Sci. Rep..

[28] Formanowicz D., Rybarczyk A., Radom M., Formanowicz P. (2020). A role of inflammation and immunity in essential hypertension—modeled and analyzed using Petri nets. Int. J. Mol. Sci..

[29] Bordon J., Moškon M., Zimic N., Mraz M. (2018). Semi-quantitative modelling of gene regulatory processes with unknown parameter values using fuzzy logic and Petri nets. Fundam. Inform..

[30] Gutowska K., Kogut D., Kardynska M., Formanowicz P., Smieja J., Puszynski K. (2022). Petri nets and ODEs as complementary methods for comprehensive analysis on an example of the ATM–p53–NF-*kappa*B signaling pathways. Sci. Rep..

[31] Grunwald S., Speer A., Ackermann J., Koch I. (2008). Petri net modelling of gene regulation of the Duchenne muscular dystrophy. BioSystems.

[32] Sackmann A., Heiner M., Koch I. (2006). Application of Petri net based analysis techniques to signal transduction pathways. BMC Bioinform..

[33] Scheidel J., Lindauer K., Ackermann J., Koch I. (2015). Quasi-steady-state analysis based on structural modules and timed Petri net predict system’s dynamics: The life cycle of the insulin receptor. Metabolites.

[34] Scheidel J., Amstein L., Ackermann J., Dikic I., Koch I. (2016). In silico knockout studies of xenophagic capturing of Salmonella. PLoS Comput. Biol..

[35] Amstein L., Ackermann J., Scheidel J., Fulda S., Dikic I., Koch I. (2017). Manatee invariants reveal functional pathways in signaling networks. BMC Syst. Biol..

[36] Hannig J., Giese H., Schweizer B., Amstein L., Ackermann J., Koch I. (2019). isiKnock: In silico knockouts in signaling pathways. Bioinformatics.

[37] Schuster S., Fell D.A., Dandekar T. (2000). A general definition of metabolic pathways useful for systematic organization and analysis of complex metabolic networks. Nat. Biotechnol..

[38] Sackmann A., Formanowicz D., Formanowicz P., Koch I., Blazewicz J. (2007). An analysis of the Petri net based model of the human body iron homeostasis process. Comput. Biol. Chem..

[39] Azkur A.K., Akdis M., Azkur D., Sokolowska M., van de Veen W., Brüggen M.C., O’Mahony L., Gao Y., Nadeau K., Akdis C.A. (2020). Immune response to SARS-CoV-2 and mechanisms of immunopathological changes in COVID-19. Allergy.

[40] von Wasielewski S., Franklin J., Fischer R., Hübner K., Hansmann M.L., Diehl V., Georgii A., von Wasielewski R. (2003). Nodular sclerosing Hodgkin disease: New grading predicts prognosis in intermediate and advanced stages. Blood J. Am. Soc. Hematol..

[41] Donnadieu E., Dupré L., Pinho L.G., Cotta-de Almeida V. (2020). Surmounting the obstacles that impede effective CAR T cell trafficking to solid tumors. J. Leukoc. Biol..

[42] Balazki P., Lindauer K., Einloft J., Ackermann J., Koch I. (2015). MONALISA for stochastic simulations of Petri net models of biochemical systems. BMC Bioinform..

[43] Starke P.H. (1990). Analyse von Petri-Netz-Modellen.

[44] Starke P.H., Roch S. INA: Integrated Net Analyzer. Reference Manual 1992. https://www2.informatik.hu-berlin.de/~starke/ina.html.

[45] von Andrian U.H., Mempel T.R. (2003). Homing and cellular traffic in lymph nodes. Nat. Rev. Immunol..

[46] Lennet K., Kikuchi M., Sato E., Suchi T., Stansfeld A.G., Feller A.C., Hansmann M.L., Müller-Hermelink H.K., Gödde-Salz E. (1985). HTLV-positive and -negative T-cell lymphomas. Morphological and immunohistochemical differences between European and HTLV-positive Japanese T-cell lymphomas. Int. J. Cancer.

[47] Cogliatti S.B., Schmid U., Schumacher U., Eckert F., Hansmann M.L., Hedderich J., Takahashi H., Lennert K. (1991). Primary B-cell gastric lymphoma: A clinicopathological study of 145 patients. Gastroenterology.

[48] Küppers R., Klein U., Hansmann M.L., Rajewsky K. (1999). Cellular origin of human B-cell lymphomas. N. Engl. J. Med..

[49] Diehl V., Sextro M., Franklin J., Hansmann M., Harris N., Jaffe E., Poppema S., Harris M., Franssila K., van Krieken J. (1999). Clinical presentation, course, and prognostic factors in lymphocyte-predominant Hodgkin’s disease and lymphocyte-rich classical Hodgkin’s disease: Report from the European task force on lymphoma project on lymphocyte-predominant Hodgkin’s disease. J. Clin. Oncol..

[50] Hannig J., Schäfer H., Ackermann J., Hebel M., Schäfer T., Döring C., Hartmann S., Hansmann M.L., Koch I. (2020). Bioinformatics analysis of whole slide images reveals significant neighborhood preferences of tumor cells in Hodgkin lymphoma. PLoS Comput. Biol..

[51] Roncador G., Verdes-Montenegro J.F.G., Tedoldi S., Paterson J.C., Klapper W., Ballabio E., Maestre L., Pileri S., Hansmann M.L., Piris M.A. (2007). Expression of two markers of germinal center T cells (SAP and PD-1) in angioimmunoblastic T-cell lymphoma. Haematologica.

[52] Schäfer T., Schäfer H., Schmitz A., Ackermann J., Dichter N., Döring C., Hartmann S., Hansmann M.L., Koch I. (2013). Image database analysis of Hodgkin lymphoma. Comput. Biol. Chem..

[53] Schäfer H., Schäfer T., Ackermann J., Dichter N., Döring C., Hartmann S., Hansmann M.L., Koch I. (2016). CD30 cell graphs of Hodgkin lymphoma are not scale-free–an image analysis approach. Bioinformatics.

[54] Oswald M.S., Wurzel P., Hansmann M.L. (2019). 3D analysis of morphological alterations of the fibroblastic reticular cells in reactive and neoplastic human lymph nodes. Acta Histochem..

[55] Thomos M., Wurzel P., Scharf S., Koch I., Hansmann M.L. (2020). 3D investigation shows walls and wall-like structures around human germinal centres, probably regulating T-and B-cell entry and exit. PLoS ONE.

[56] Donnadieu E., Reisinger K.B., Scharf S., Michel Y., Bein J., Hansen S., Loth A.G., Flinner N., Hartmann S., Hansmann M.L. (2020). Landscape of T follicular helper cell dynamics in human germinal centers. J. Immunol..

[57] Wurzel P., Ackermann J., Schäfer H., Scharf S., Hansmann M.L., Koch I. (2021). Detection of follicular regions in actin-stained whole slide images of the human lymph node by shock filter. Biol. Chem..

[58] Lumer L., Wurzel P., Scharf S., Schäfer H., Ackermann J., Koch I., Hansmann M.L. (2021). 3D connectomes of reactive and neoplastic CD30 positive lymphoid cells and surrounding cell types. Acta Histochem..

[59] Hartmann S., Scharf S., Steiner Y., Loth A.G., Donnadieu E., Flinner N., Poeschel V., Angel S., Bewarder M., Bein J. (2021). Landscape of 4D cell interaction in Hodgkin and non-Hodgkin lymphomas. Cancers.

[60] Schuster S., Hilgetag C. (1994). On elementary flux modes in biochemical reaction systems at steady state. J. Biol. Syst..

[61] Schuster S., Dandekar T., Fell D.A. (1999). Detection of elementary flux modes in biochemical networks: A promising tool for pathway analysis and metabolic engineering. Trends Biotechnol..

[62] Stelling J., Klamt S., Bettenbrock K., Schuster S., Gilles E.D. (2002). Metabolic network structure determines key aspects of functionality and regulation. Nature.

[63] Heiner M., Koch I., Will J. (2004). Model validation of biological pathways using Petri nets—Demonstrated for apoptosis. BioSystems.

[64] Koch I., Junker B.H., Heiner M. (2005). Application of Petri net theory for modelling and validation of the sucrose breakdown pathway in the potato tuber. Bioinformatics.

[65] Keating S.M., Waltemath D., König M., Zhang F., Dräger A., Chaouiya C., Bergmann F.T., Finney A., Gillespie C.S., Helikar T. (2020). SBML Level 3: An extensible format for the exchange and reuse of biological models. Mol. Syst. Biol..

[66] Pikor N.B., Mörbe U., Lütge M., Gil-Cruz C., Perez-Shibayama C., Novkovic M., Cheng H.W., Nombela-Arrieta C., Nagasawa T., Linterman M.A. (2020). Remodeling of light and dark zone follicular dendritic cells governs germinal center responses. Nat. Immunol..

[67] Victora G.D., Nussenzweig M.C. (2012). Germinal centers. Annu. Rev. Immunol..

[68] Baldan P., Cocco N., Marin A., Simeoni M. (2010). Petri nets for modelling metabolic pathways: A survey. Nat. Comput..

[69] Chaouiya C. (2007). Petri net modelling of biological networks. Briefings Bioinform..

[70] Tay M.Z., Poh C.M., Rénia L., MacAry P.A., Ng L.F. (2020). The trinity of COVID-19: Immunity, inflammation and intervention. Nat. Rev. Immunol..

[71] Doan T.A., Forward T., Tamburini B.A.J. (2022). Trafficking and retention of protein antigens across systems and immune cell types. Cell. Mol. Life Sci..

[72] Wagner P., Strodthoff N., Wurzel P., Marban A., Scharf S., Schäfer H., Seegerer P., Loth A., Hartmann S., Klauschen F. (2022). New definitions of human lymphoid and follicular cell entities in lymphatic tissue by machine learning. Sci. Rep..

[73] Vyas J.M., Van der Veen A.G., Ploegh H.L. (2008). The known unknowns of antigen processing and presentation. Nat. Rev. Immunol..

[74] Hendrikx J.J., Haanen J.B., Voest E.E., Schellens J.H., Huitema A.D., Beijnen J.H. (2017). Fixed dosing of monoclonal antibodies in oncology. Oncologist.

[75] Perez-Andres M., Paiva B., Nieto W.G., Caraux A., Schmitz A., Almeida J., Vogt Jr R., Marti G., Rawstron A., Van Zelm M. (2010). Human peripheral blood B-cell compartments: A crossroad in B-cell traffic. Cytom. Part B Clin. Cytom..

[76] Lanzavecchia A., Sallusto F. (2001). Regulation of T cell immunity by dendritic cells. Cell.

[77] Von Andrian U.H., Mackay C.R. (2000). T-cell function and migration—Two sides of the same coin. N. Engl. J. Med..

[78] Xu W., Joo H., Clayton S., Dullaers M., Herve M.C., Blankenship D., De La Morena M.T., Balderas R., Picard C., Casanova J.L. (2012). Macrophages induce differentiation of plasma cells through CXCL10/IP-10. J. Exp. Med..

[79] LeBien T.W., Tedder T.F. (2008). B lymphocytes: How they develop and function. Blood.

[80] McHeyzer-Williams L.J., McHeyzer-Williams M.G. (2005). Antigen-specific memory B cell development. Annu. Rev. Immunol..

[81] Qi H., Egen J.G., Huang A.Y.C., Germain R.N. (2006). Extrafollicular activation of lymph node B cells by antigen-bearing dendritic cells. Science.

[82] Gonzalez S.F., Degn S.E., Pitcher L.A., Woodruff M., Heesters B.A., Carroll M.C. (2011). Trafficking of B cell antigen in lymph nodes. Annu. Rev. Immunol..

[83] Vinuesa C.G., Sanz I., Cook M.C. (2009). Dysregulation of germinal centres in autoimmune disease. Nat. Rev. Immunol..

[84] Chen X., Zheng Y., Liu S., Yu W., Liu Z. (2022). CD169+ subcapsular sinus macrophage-derived microvesicles are associated with light zone follicular dendritic cells. Eur. J. Immunol..

[85] Van Zelm M.C., Szczepanski T., Van Der Burg M., Van Dongen J.J. (2007). Replication history of B lymphocytes reveals homeostatic proliferation and extensive antigen-induced B cell expansion. J. Exp. Med..

[86] Sixt M., Kanazawa N., Selg M., Samson T., Roos G., Reinhardt D.P., Pabst R., Lutz M.B., Sorokin L. (2005). The conduit system transports soluble antigens from the afferent lymph to resident dendritic cells in the T cell area of the lymph node. Immunity.

[87] Manolova V., Flace A., Bauer M., Schwarz K., Saudan P., Bachmann M. (2008). Nanoparticles target distinct dendritic cell populations according to their size. Eur. J. Immunol..

[88] Batista F.D., Harwood N.E. (2009). The who, how and where of antigen presentation to B cells. Nat. Rev. Immunol..

[89] Gray E.E., Cyster J.G. (2012). Lymph node macrophages. J. Innate Immun..

[90] Stewart I., Radtke D., Phillips B., McGowan S.J., Bannard O. (2018). Germinal center B cells replace their antigen receptors in dark zones and fail light zone entry when immunoglobulin gene mutations are damaging. Immunity.

[91] Zhang Y., Garcia-Ibanez L., Ulbricht C., Lok L.S., Pike J.A., Mueller-Winkler J., Dennison T.W., Ferdinand J.R., Burnett C.J., Yam-Puc J.C. (2022). Recycling of memory B cells between germinal center and lymph node subcapsular sinus supports affinity maturation to antigenic drift. Nat. Commun..

[92] Xia Z., Triffitt J.T. (2006). A review on macrophage responses to biomaterials. Biomed. Mater..

[93] Louie D.A.P., Liao S. (2019). Lymph node subcapsular sinus macrophages as the frontline of lymphatic immune defense. Front. Immunol..

[94] Martinez-Pomares L., Gordon S. (2012). CD169+ macrophages at the crossroads of antigen presentation. Trends Immunol..

[95] Brown F.D., Turley S.J. (2015). Fibroblastic reticular cells: Organization and regulation of the T lymphocyte life cycle. J. Immunol..

[96] Lanzavecchia A. (1985). Antigen-specific interaction between T and B cells. Nature.

[97] Cahalan M.D., Parker I. (2005). Close encounters of the first and second kind: T–DC and T–B interactions in the lymph node. Semin. Immunol..

[98] Biram A., Davidzohn N., Shulman Z. (2019). T cell interactions with B cells during germinal center formation, a three-step model. Immunol. Rev..

[99] Allen C.D.C., Okada T., Tang H.L., Cyster J.G. (2007). Imaging of germinal center selection events during affinity maturation. Science.

[100] Tangye S.G., Good K.L. (2007). Human IgM+ CD27+ B cells: Memory B cells or “memory” B cells?. J. Immunol..

[101] Fooksman D.R., Schwickert T.A., Victora G.D., Dustin M.L., Nussenzweig M.C., Skokos D. (2010). Development and migration of plasma cells in the mouse lymph node. Immunity.

[102] Zotos D., Tarlinton D.M. (2012). Determining germinal centre B cell fate. Trends Immunol..

[103] Davies D.R., Padlan E.A., Sheriff S. (1990). Antibody-antigen complexes. Annu. Rev. Biochem..

